# An examination of the Northern Hemisphere mid-latitude storm track interannual variability simulated by climate models—sensitivity to model resolution and coupling

**DOI:** 10.1007/s00382-018-4378-x

**Published:** 2018-08-04

**Authors:** Xuelei Feng, Bohua Huang, George Tintera, Baohua Chen

**Affiliations:** 1 Center for Climate Physics, Institute for Basic Science, Pusan, 46241 South Korea; 2 Pusan National University, Pusan, 46241 South Korea; 3 Department of Atmospheric, Oceanic, and Earth Sciences, George Mason University, Fairfax, VA 22030 USA; 4 Center for Ocean-Land-Atmosphere Studies, George Mason University, Fairfax, VA 22030 USA; 5 Department of Mathematics and Statistics, Texas A&M University-Corpus Christi, Corpus Christi, TX 78412 USA

## Abstract

The model fidelity in simulating the Northern Hemisphere storm track interannual variability and the connections of this variability to the low frequency atmospheric variations and oceanic variations are examined based on the atmospheric European Centre for Medium-Range Weather Forecasts (ECMWF) model and coupled NCAR Community Climate System Model (CCSM) systems at different horizontal resolutions. The atmospheric general circulation model (AGCM) runs are forced by observed sea surface temperatures (SST) with varying atmospheric resolutions, while the coupled general circulation model (CGCM) runs have a fixed atmospheric resolution but varying oceanic resolutions. The phases, between the North Pacific (NP) and North Atlantic (NA) sectors, of the simulated hemisphere-scale Empirical Orthogonal Function (EOF) modes of the storm track fluctuations change with the model resolution, suggesting the storm track variability in NP and NA basins are largely independent. The models can qualitatively reproduce the basin-scale EOFs of both NP and NA storm track variability. These EOFs are not sensitive to either atmospheric or oceanic model horizontal resolutions, but their magnitudes from the CGCM runs are substantially underestimated. The storm track variations over NP basin are hybrid of internal atmospheric variations and external forcing from the underlying conditions, but the fluctuations over the NA basin are merely atmospheric internal variability. The NP storm track variability from SST forcing accounts for 4.4% of the total variance in observations, while it only has less than 2% of the total in all AGCM simulations. The external forcing to the storm track variations is more realistically reproduced in the higher atmospheric resolution runs. The air–sea coupling makes the SST feedbacks to the atmospheric internal variability, absent in the atmospheric ECMWF model hindcasts, emerge in the coupled CCSM simulations.

**Electronic supplementary material:**

The online version of this article (10.1007/s00382-018-4378-x) contains supplementary material, which is available to authorized users.

## Introduction

The poleward transports of heat and momentum are enormous by the midlatitude atmospheric weather systems. These transient eddy transports are critical in maintaining the climatology of the temperature distribution and other fundamental features of the atmospheric general circulation. Mid-latitude storms are also an energy source of the lower-frequency fluctuations of the extratropical circulation through mean-wave conversions. From the synoptic perspective, storm tracks are regions where synoptic-scale cyclones are prevalent. These storm tracks usually lie in the mid-latitudes and generate extreme precipitation climatology (Hawcroft et al. 2012; Pfahl and Wernli 2012) and strong winds, potentially resulting in storm surges, and thus are the causes of the major natural hazards of these regions (Leckebusch and Ulbrich 2004; Pinto et al. 2007; Schwierz et al. 2010). Blackmon (1976) showed that in the Northern Hemisphere (NH) there are two variability peaks of 500-hPa geopotential height fields over the mid-latitude oceanic regions, which correspond closely to the locations of maximum synoptic-scale cyclone activity. Interestingly, these two peak regions of cyclone activity are located over the two major oceanic frontal zones, the Kuroshio and Gulf Stream extensions over the mid-latitude oceans, and form two distinct tracks: the North Pacific (NP) branch and North Atlantic (NA) branch (Graff and LaCasce 2012).

In the previous studies, the lower frequency variability of the storm tracks is generally analyzed using the monthly variance field of the synoptic variations. For instance, Lau (1988) documented the dominant patterns of mid-latitude storm-track variability on time scales of a month or longer and identified the boreal winter pattern using the empirical orthogonal function (EOF) decomposition on the hemispheric fields of the operational atmospheric analysis data produced by the National Meteorological Center of US. The first two leading modes of storm track variability in both the NP and NA sectors respectively represent monopole patterns overlaid on the major storm track background, and dipole patterns straddled over the climatological mean storm tracks. The mono-sign structure is dominated by the center in the NP sector, while the dipole structure is dominated by the one in the NA sector. Also examining the EOF-based hemispheric storm track modes, Chang and Fu (2002) suggest that the strengths of the two storm tracks can fluctuate coherently on interannual time scales. They also suggest the existence of a hemisphere-scale pattern of NH storm track variability, due to the fact that the interannual and month-to-month variations of the NP and NA storm tracks are found to be significantly correlated in the winter season based on the National Centers for Environmental Prediction–National Center for Atmospheric Research (NCEP–NCAR) reanalysis datasets. By revealing the high correlations of high-passed 250-hPa meridional wind variances between the NP and Atlantic from reanalysis and observations, Chang’s (2004) results support the idea of a hemispheric pattern of storm track variability. More recently, Wettstein and Wallace (2010) have performed the hemispheric EOF analyses on both hemispheric and sectorial domains using the ERA-40 monthly variance fields of the high-pass filtered winds. They also reveal the mono-sign and dipole structures in their leading EOF modes of the hemispheric fields. Their further analyses within the NP and Atlantic basins, however, suggest that the storm-track variability of the two Northern Hemisphere sectors appears to be largely independent from each other.

Lau (1988) further examines the connections between the leading modes of the two NH sectorial storm track variability and the atmospheric lower-frequency teleconnection patterns by correlating the corresponding leading principal components (PCs) of the storm track modes to the major teleconnection pattern indices defined in Wallace and Gutzler (1981). Detailed connections among storm track variability modes, atmospheric teleconnection patterns and the upper-tropospheric jet streams are also discussed in Wettstein and Wallace (2010). The low-frequency variations of the storm tracks show similar patterns in two sectors. In general, the storm track variability exhibits not only a strengthening or weakening at the locations of climatological center (pulsing) but also latitudinal shifting associated with atmospheric lower frequency variability (Lau 1988; Yang and Chang 2006, 2007). Particularly, a major variation in the NP is in response to the atmospheric teleconnection pattern of the El Niño–Southern Oscillation (ENSO) cycle associated with atmosphere–ocean interaction (Grise et al. 2013). During El Niño years, the Pacific storm track shifts equatorward and downstream (Straus and Shukla 1997; Zhang and Held 1999; Eichler and Higgins 2006), while in La Niña years it shifts toward the opposite direction (Yang et al. 2015).

There have been many previous studies regarding the midlatitude storm tracks. However, few studies have been done to systematically examine the model resolution influences in simulating the storm track variations. In this article, we will assess the sensitivity of the simulated storm track variability to the model horizontal resolution and the air–sea coupling based on the atmospheric and coupled model frameworks. In particular, we firstly examine the connections of the storm track variations between NP and NA basins by varying the atmospheric model resolutions. Secondly, we reaffirm the leading EOF modes of the storm track variations in each basin, and reassess their associations with the major low-frequency atmospheric and oceanic fluctuations. By comparing the oceanic low frequency patterns linked to storm track variations between atmospheric and coupled model runs, we then identify the nature (e.g., internal atmospheric process or external forcing) of the storm track activities. Meanwhile the sensitivity of the simulated phenomena to model resolutions and air–sea coupling, as well as the model fidelity in reproducing the storm track variability are evaluated.

Outputs from atmospheric European Center for Medium-Range Weather Forcasts (ECMWF) Integrated Forecasting System (IFS) hindcasts at the resolutions of T159, T511, and T1279, as well as those from the Community Climate System Model (CCSM) HRC06 runs are used for the study of low-frequency variations of the Northern Hemisphere storm tracks in this paper. The band-pass filtered variance proves to be a useful measure of storm track variability (Chang 2009). Following Wettstein and Wallace (2010), we use the high frequency band-pass filtered variance of the meridional wind component as a storm-track indicator. For this purpose, the daily 250 hPa meridional winds from the model outputs, and from the ERA-40 atmospheric reanalysis are firstly high-pass filtered. The filter used in this study is a 13th order Butterworth filter with a 50% cutoff signal at a period of 6 days. Data from ERA-40, covering the period of 1961–2001, are used to verify the set of atmospheric model runs. The monthly averages of the high-pass filtered wind squared ($\stackrel{-}{{v}^{\text{'}}{v}^{\text{'}}}$, denoted as *vv*_250_ hereafter) are used to measure the monthly intensity of the storm track strength. The high-pass filtered 300 hPa meridional winds (*vv*_300_) are used in Wettstein and Wallace (2010). The *vv*_250_ is used in this study because the 300 hPa winds are not part of the model outputs.

The remainder of this paper is structured as follows. Section 2 introduces data and model configuration. Section 3 analyzes the hemispheric patterns of Northern Hemisphere storm track activities from ECMWF atmospheric model hindcasts. The sectorial patterns from atmospheric model hindcasts, as well as their connections with atmospheric and oceanic low-frequency variability are described in Sect. 4. The identical analysis with that in Sect. 4 is applied to coupled model simulations and discussed in Sect. 5. The summary is provided in Sect. 6.

## Simulations and observed data

We use two sets of existing climate model simulations to examine the interannual variability of the Northern Hemisphere storm tracks. One set is based on the European Centre for Medium-Range Weather Forecasts (ECMWF) Integrated Forecast System (IFS) atmospheric general circulation model (AGCM) forced with observed sea surface temperatures (SST) (the Athena runs). The AGCM runs use different atmospheric resolutions, the highest at 16 km. The other set is based on the coupled Community Climate System Model (CCSM) simulations with oceanic general circulation model (OGCM) resolutions at 100 and 10 km. These simulations as well as the observed datasets used in this study are introduced below.

### Athena atmospheric simulations

The project Athena is an international collaborative project of five institutions in response to the call of a revolution in seamless weather and climate modeling made at the World Modeling Summit, held in May 2008 in Reading, UK (Shukla and Hagedorn 2009). It brought together an international team of climate-weather modelers and high-end computing experts to test whether representing mesoscale and sub-synoptic atmospheric processes in climate models improves climate simulation and prediction. For a part of Project Athena, numerical simulations were carried out with the European Centre for Medium-Range Weather Forecasts (ECMWF) Integrated Forecast System (IFS), a hydrostatic spectral model used for operational forecasting. Multiple simulations were designed to accurately resolve synoptic and mesoscale atmospheric phenomena by increasing weather and climate model resolution. The Athena supercomputer, a dedicated computing resource, operated by the University of Tennessee’s National Institute for Computational Science (NICS) and hosted by Oak Ridge National Laboratory (ORNL), is used to make different experiments. More details about Athena project are introduced by Kinter et al. (2013).

ECMWF IFS hindcasts done in Project Athena are comprised of 13-month long integrations starting on 1 November of each year for four different resolutions, which were named according to the cutoff wave number used in the spherical harmonics expansion, T159, T511, T1279, and T2047, corresponding to 320, 1024, 2560, and 4096 grid points along the equator in order (Dirmeyer et al. 2012). The first month spin-up outputs are discarded in our analysis. The associated model configurations are depicted in Feng et al. (2017). Most of the T511, T1279, and T2047 fields are truncated to T159 and interpolated to a corresponding N80 grid. Using the truncated outputs, we can directly compare features at the same spatial scales from different model runs. The T159, T511 and T1279 runs were conducted for the years 1961–2007, while the T2047 run was carried out only for the years 1989–2007. To maintain consistency across data sets, we have not included the T2047 run in this analysis.

### CCSM3.5 simulations

Project Athena examined the AGCM simulated storm track variations. However, the oceanic component and the atmosphere–ocean coupling may also have important effects on these processes. In order to further examine the roles of the oceanic settings and the coupling in storm track variations, a pre-release of NCAR CCSM version 4.0 is introduced in this study. The CCSM is a state-of-the-art coupled climate model consisting of atmosphere, ocean, land, and sea ice components. Details of the model configuration and an overview of the simulated climate for this set of experiments are given in Kirtman et al. (2012). Briefly, the atmosphere component is the Community Atmosphere Model (CAM) with 26 vertical levels in a hybrid coordinate. Its horizontal resolution is fixed at zonal grid spacing of 0.625° and meridional grid spacing of 0.5° in all experiments. The land component has the same horizontal resolution as the atmosphere component. The oceanic component has 42 levels vertically but different horizontal resolutions for different runs. The low and high-resolution runs are referred to as LRC01 and HRC06 hereafter, following Kirtman et al. (2012).

### Observed dataset

ERA-40 (Uppala et al. 2005) is used for model verification in this study. It is a reanalysis of meteorological observations from September 1957 to August 2002 produced by the ECMWF. The data are on spectral format with the resolution of T85 at a Gaussian grid with 256 × 128 points globally. Variables adopted include the meridional wind and geopotential height at 250 hPa level.

## The relationship between NP and NA sectors based on AGCM runs

**Fig. 1:**
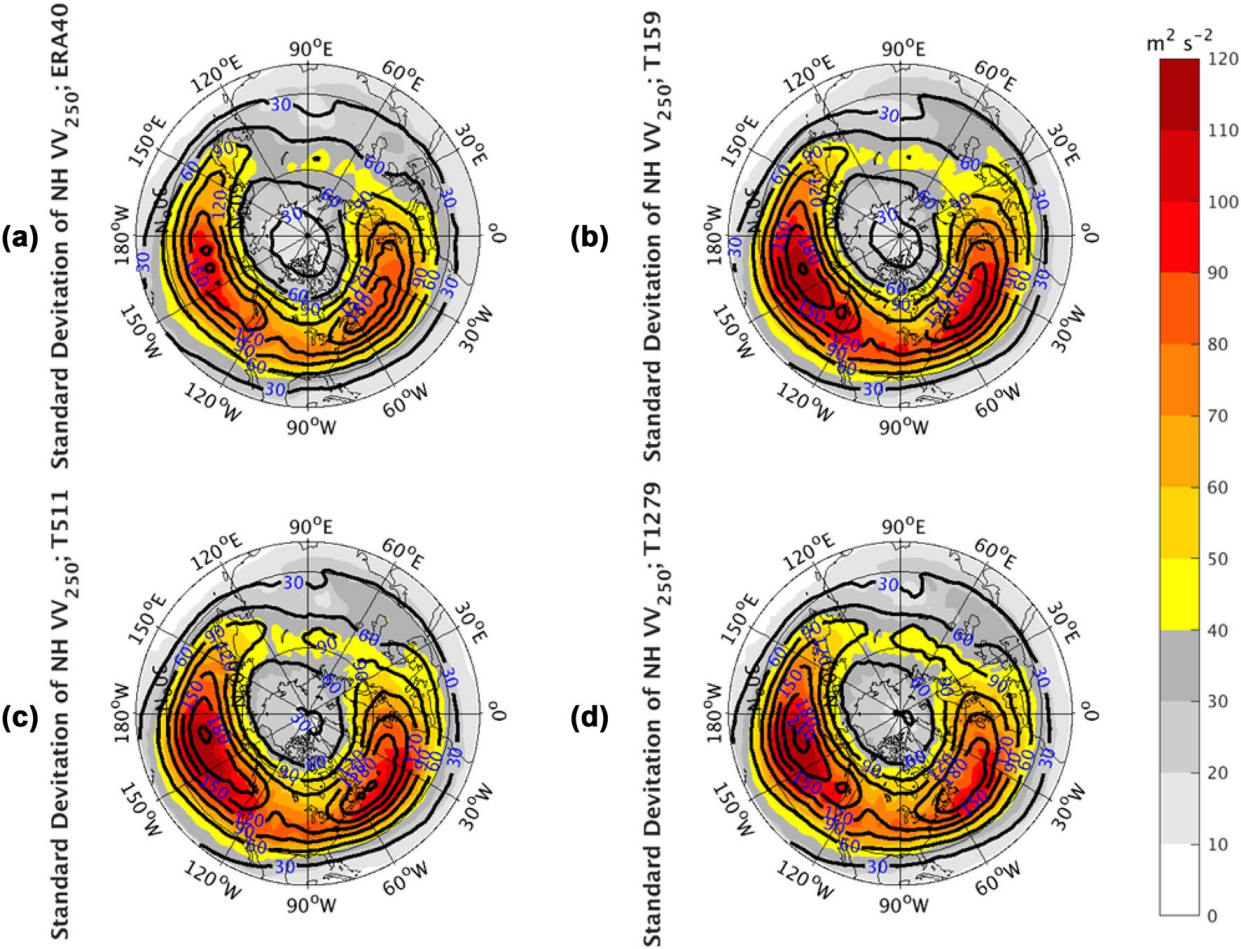
The climatology (contours) and standard deviation (shaded) of the NH *vv*_250_ (1960.12–2001.11)

In this section, we explore the relationships of storm track variations between the NP and NA basins. Prior to the variability study, the IFS model simulated *vv*_250_ climatology (contours) and standard deviation (shaded) are compared with the observations (Fig. 1). The observed panel (Fig. 1a) shows a band of mean state storms stands out in the mid-latitudes with two pronounced centers over the NP and NA basins. The storm track standard deviation pattern can well resemble the climatology pattern. The simulations at all three resolutions (Fig. 1b–d) are able to reproduce the broad structures, including the two maxima of storm tracks over the NP and NA basins, the weaker intensity zone between these two maxima, and their realistic positions. The simulated intensities of climatology and standard deviation are also comparable to the observed, even though there are slight overestimates of the two maxima over the oceans. Both the intensity and structure of the NH storm tracks are not notably sensitive to the model horizontal resolutions.

**Fig. 2:**
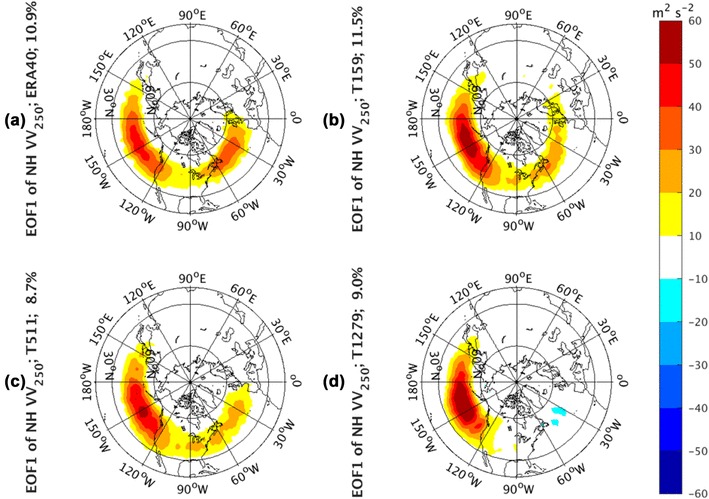
EOF1 of Northern Hemisphere monthly averaged *vv*_250_ from the ERA-40 reanalysis for 1960.12–2001.11 in **a**, as well as Athena IFS simulations at T159 in **b**, T511 in **c**, and T1279 in **d**

**Fig. 3:**
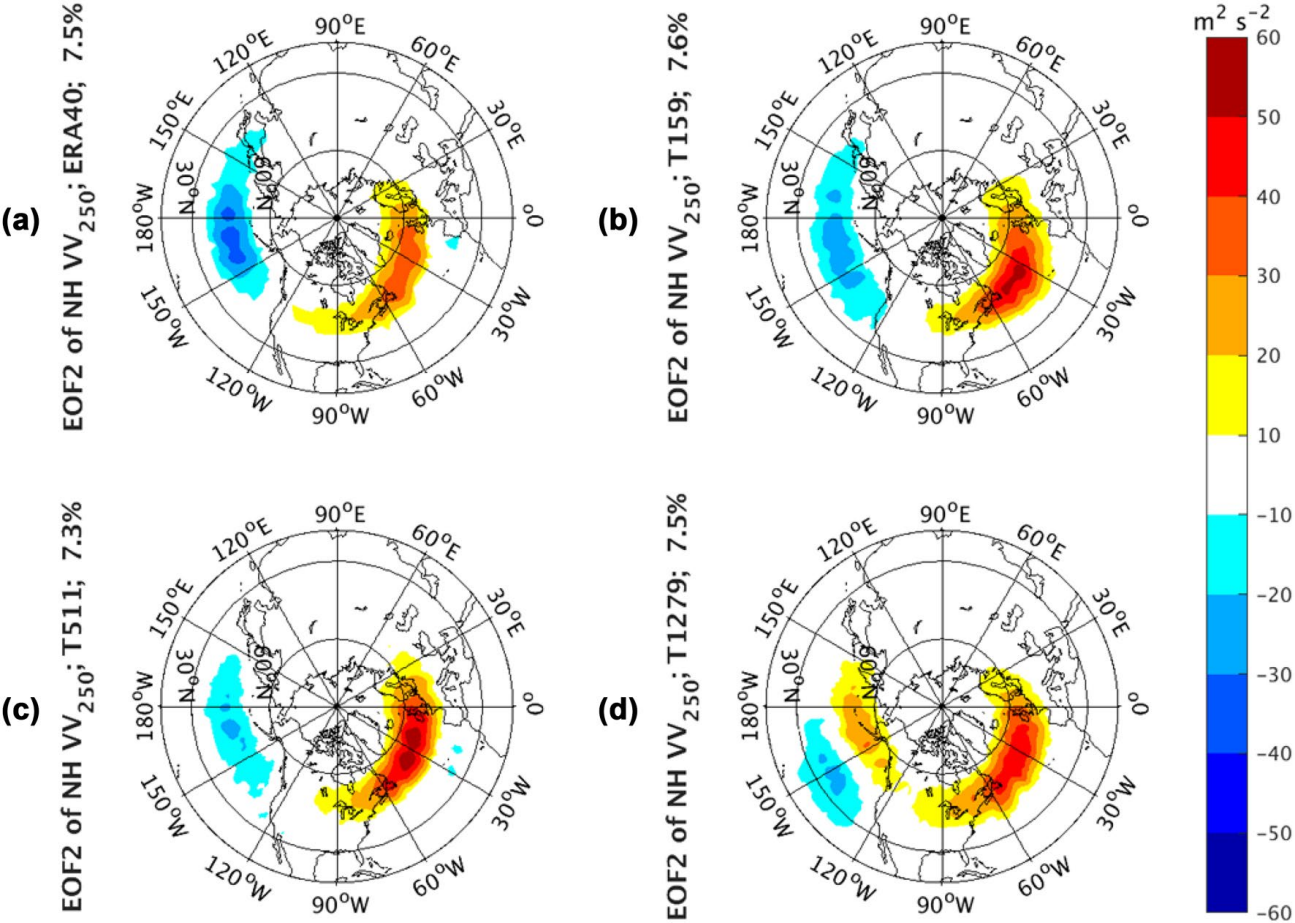
EOF2 of Northern Hemisphere monthly averaged *vv*_250_ from the ERA-40 reanalysis for 1960.12–2001.11 in **a**, as well as Athena IFS simulations at T159 in **b**, T511 in **c**, and T1279 in **d**

The EOF analysis to *vv*_250_ is applied to examine the storm track activities of the whole NH domain. The North et al.’s (1982) rule of thumb is adopted to test the uniqueness of the EOF modes. According to the Eq. (24) of North et al. (1982), we found the numbers of the distinguishable modes of NH *vv*_250_ for T159, T1511, and T1279 are 2, 6, and 2, respectively. For consistence, only the first two leading EOF modes of *vv*_250_ from all runs are investigated and demonstrated in the shadings in Figs. 2 and 3. In addition, the EOF analysis is also performed on the ERA-40 *vv*_250_ for verification. ERA40 has three distinguishable EOF modes. The first EOF mode (EOF1) of *vv*_250_ from ERA-40 reanalysis is demonstrated in Fig. 2a with 10.9% of the total variance explained. The counterparts based on the outputs from the Athena hindcast are generated as well and exhibited in the rest of the panels of Fig. 2. The panel for EOF1 from reanalysis data shows the strengthening and weakening of the two storm track centers (shading) exerted on the two climatological-mean storm track branches (contours) over the oceans. This is very similar to EOF1 of the variance of high-pass filtered ERA-40 daily 300 hPa meridional wind (*vv*_300_) shown in Fig. 1 (upper left panel) of Wettstein and Wallace (2010). As we have mentioned above, the strengthening and weakening of the storm tracks at their climatological center is usually characterized as the pulsing signal. The spatial pattern of this mode suggests that, as the leading mode of variability, the storm tracks in these two basins pulse (strengthen and weaken) in phase in time.

The leading EOF modes from AGCM simulations show quite different patterns. The pattern from the lowest resolution (T159) simulation (Fig. 2b) is consistent with the ERA-40 reanalysis and able to capture the gross features of these in-phase relationships between the two basins. In addition, the variance explained in T159 (11.0%) is comparable to that from the reanalysis (10.9%). However, the pulsing signal in the Atlantic sector vanishes gradually (Fig. 2c, d) with resolution increases, indicating that the dominant variability is oriented in the Pacific sector and that this in-phase relationship is not steady. As a result, the leading EOF pattern in the T511 and T1279 simulations are characterized by the pulsing of the NP storm tracks only, with little corresponding variations in the NA sector.

The second EOFs from the NH analysis are shown in Fig. 3. The panel order of this figure and the rest figures associated with the Athena database is the same as that in Fig. 1. The observed EOF2 in Fig. 3a represents a seesaw between the strength of the Pacific and Atlantic storm tracks, i.e., their strength is out of phase (one strong, the other weak). This is reminiscent of the EOF2 of ERA-40 *vv*_300_ in Wettstein and Wallace (2010) (the left bottom panel of their Fig. 1). This mode from observations explains 7.6% of the total variance. The IFS T159 simulation (Fig. 3b) again yields the most consistent pattern with the seesaw relations from ERA-40 and explains the percentage of total variance (7.7%) very closely to the observed one (7.6%). The T511 and T1279 simulations have an explained variance of 7.1% for the former and 7.4% for the latter. Although the anomalies centered over the NA storm track are quite robust, the opposite-sign center over the NP weakens as the model resolution increases (Fig. 3c, d). In T1279 run (Fig. 3d), the signal in the Pacific turns to latitudinal shifting from pulsing. That indicates the EOF2 mainly characterizes the pulsing in the NA sector in T511 and T1279, with little connections to the NP. EOF1 and EOF2 of NH storm track variability both suggest that the coherent pulsing signals only appear in the relatively low-resolution model simulations. As the model resolution increases, the pulsing occurs largely independently in the NP and the NA basins, with the former accounting for more variance. This independence can also be detected in the NH EOF correlations between ERA40 and Athena runs (Table 1). The EOFs from Athena hindcasts are all highly correlated with the corresponding ones from ERA40, suggesting that the models reproduce the patterns of the storm track variability well. However, it is noticeable that the NH case of T1279 shows lower correlation values with the observations, consistent with the fact that the NP and NA centers seem to be more independent of each other and do not show the in-phase/out-of-phase tendency that appear in the other runs and the observations. The in-phase/out-of-phase relationship is controversial in the literature. The rotated EOF analyses are also performed on the Northern Hemispheric basin. The first two leading rotated EOFs (Supplementary Figs. 1–2) are all confined to the individual NA or NP basin, which supports that the storm track interannual variability is largely independent.

**Table 1:** Correlation coefficients of leading EOFs (upper part) and leading PCs (lower part) between ERA40 and Athena runs

	T159	T511	T1279
EOF			
NH			
EOF1	**0.88**	**0.91**	**0.65**
EOF2	**0.88**	**0.89**	**0.54**
NP			
EOF1	**0.92**	**0.95**	**0.97**
EOF2	**0.91**	**0.94**	**0.97**
NA			
EOF1	**0.98**	**0.97**	**0.98**
EOF2	**0.91**	**0.96**	**0.83**
PC			
NH			
PC1	0.04	0.04	**0.10**
PC2	0.03	0.01	0.02
NP			
PC1	0.02	0.04	**0.10**
PC2	0.04	0.02	0.08
NA			
PC1	0.06	0.05	0.05
PC2	**0.10**	**0.11**	0.01

The coefficients passing the 95% significance test are in bold

## Sectorial patterns of the storm track activity: connections to the atmospheric and oceanic low-frequency variability in AGCM runs

Since the variability of the storm tracks is independent in the two sectors of the NH, we discuss it in the individual sectors separately. In this study, we follow the same approach as Chang and Fu (2002) to assign the domains of these two Northern Hemisphere sectors. The North et al.’s (1982) rule of thumb is also adopted in this section to test the distinguishable modes of the sectorial *vv*_250_. In the NP sector, ERA40, T159, T511, and T1279 have 9, 3, 1, and 3 distinguishable EOF modes, respectively. In the NA sector, they have 6, 3, 7, and 3 distinguishable modes in order. All simulations and observations have their two leading modes distinguishable except that NP T511 run has EOF2 and 3 mixed. The spatial patterns of the EOF2 and 3 from this run, however, do not show significant differences from those of other runs. Since a few EOF3 do pass the North rule, we have added a general discussion on their structures and properties in the Sect. 4.1. The emphasis of this paper is still on the properties and explanations of the two leading EOF modes.

### Leading modes in individual sectors

**Fig. 4:**
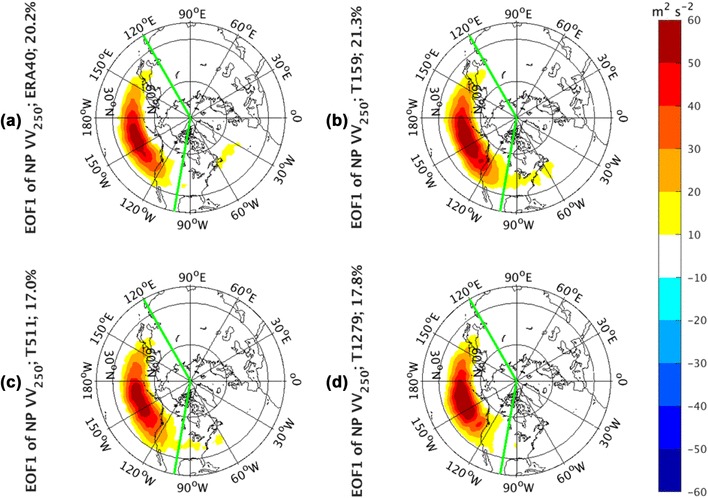
EOF1 of North Pacific sector, indicated by green lines, monthly averaged *vv*_250_ from the ERA-40 reanalysis for 1960.12–2001.11 in **a**, as well as Athena IFS simulations at T159 in **b**, T511 in **c**, and T1279 in **d**

Figure 4 demonstrates the regression pattern over the whole NH domain of *vv*_250_ to the first principal component (PC1) of *vv*_250_ calculated in the NP sector bordered by the two green lines at 120ºE and 110ºW respectively. Note that, within the bordered domain, the regression pattern is the same as the EOF pattern. The percentage of the total variance explained by this mode for the observations is 20.2%, 21.3% for IFS T159, 17.0% for IFS T511, and 17.8% for IFS T1279. The observed dominant pattern (Fig. 4a) of month-to-month storm-track variability is characterized by a pulsing on the mean state background. The large amplitudes are restricted in the regions where EOF analysis is performed, except for a small response near the center of the Atlantic storm track. All AGCM simulations (Fig. 4b–d) are able to successfully reproduce this monopole center over the mean state *vv*_250_. This variability mode is not sensitive to model resolution.

**Fig. 5:**
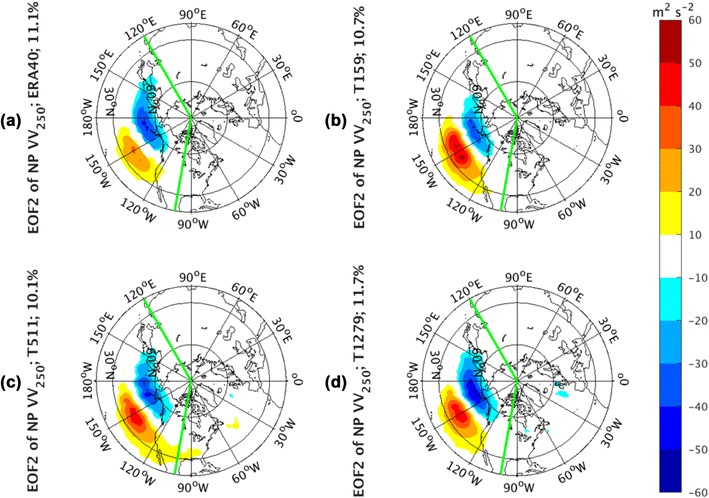
EOF2 of North Pacific sector, indicated by green lines, monthly averaged *vv*_250_ from the ERA-40 reanalysis for 1960.12–2001.11 in **a**, as well as Athena IFS simulations at T159 in **b**, T511 in **c**, and T1279 in **d**

The regression modes to PC2 of *vv*_250_ in the NP sector (Fig. 5) from both observations (Fig. 5a) and AGCM simulations are characterized by a dipole structure with its two poles straddling the axis of storm track climatology. This pattern depicts a latitudinal migration of the storm track from its mean state position. These meridional shifts of the storm track tend to be localized within the NP sector, and there is a little of downstream effects over the North American continent in T511. This second mode in the observations explains 11.1% of the total variance. All AGCM simulations from these three different model resolutions (Fig. 5b–d) are able to successfully reproduce this dipole structure. There is no apparent change with model resolution. The percentage of the total variances explained by this mode in simulations for T159 is 10.7%, for T511 10.1%, and for T1279 11.7%.

The regression structure to PC3 of NP *vv*_250_ (Supplementary Fig. 3) signatures a zonal dipole pattern, showing a longitudinal shift of the storm tracks. The explained variance of *vv*_250_ for ERA40, T159, T511, and T1279 is 9.9, 8.6, 9.9, and 8.9%, respectively. This pattern is not sensitive to the atmospheric model resolution.

**Fig. 6:**
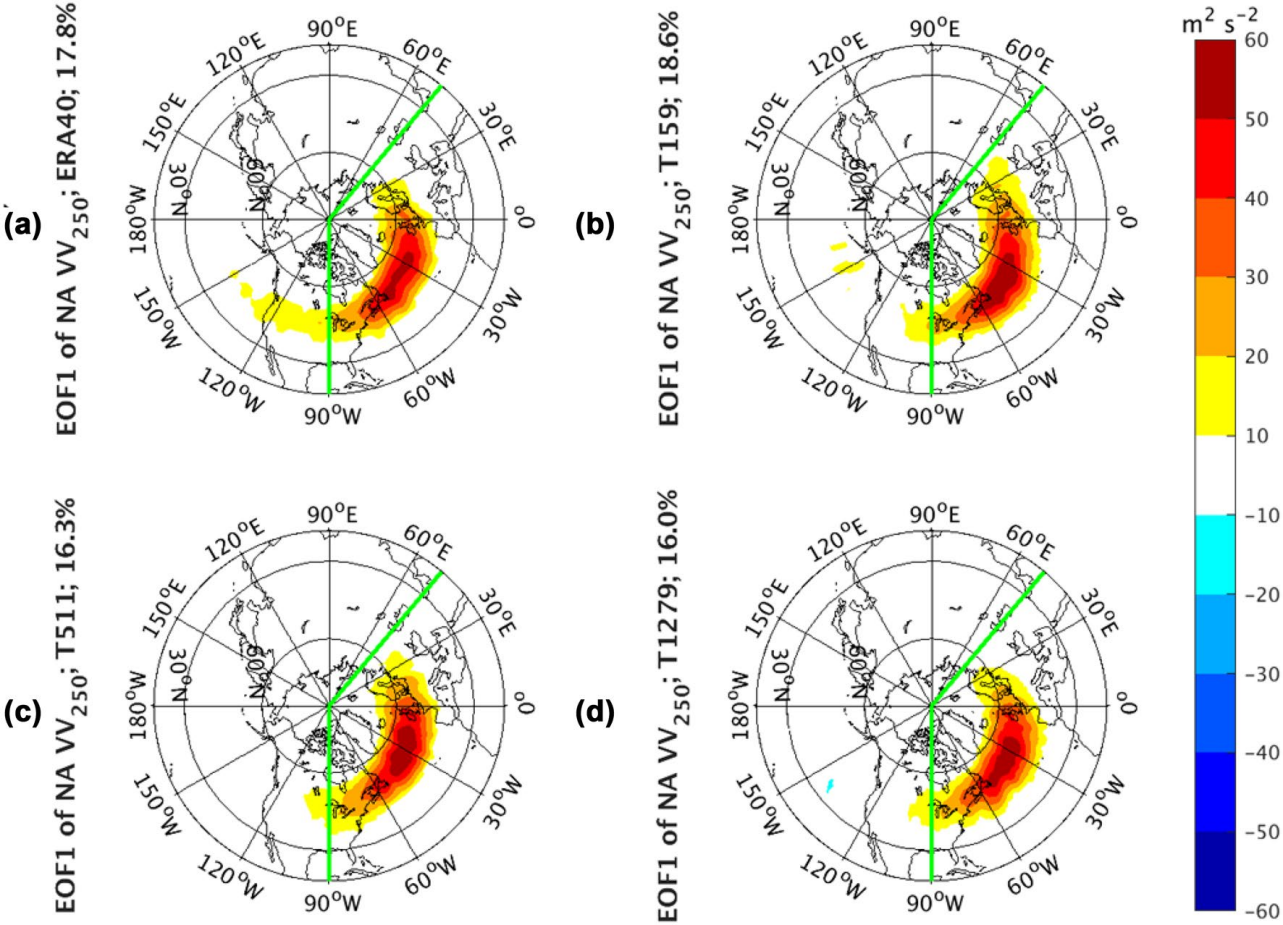
EOF1 of North Atlantic sector, indicated by green lines, monthly averaged *vv*_250_ from the ERA-40 reanalysis for 1960.12–2001.11 in **a**, as well as Athena IFS simulations at T159 in **b**, T511 in **c**, and T1279 in **d**

**Fig. 7:**
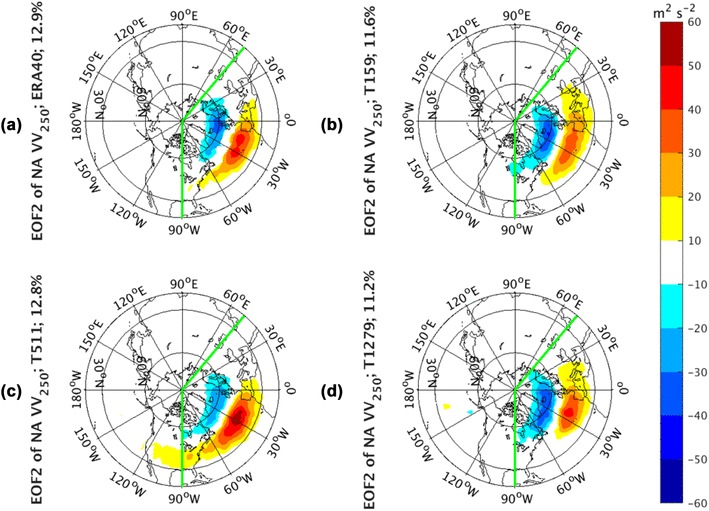
EOF2 of North Atlantic sector, indicated by green lines, monthly averaged *vv*_250_ from the ERA-40 reanalysis for 1960.12–2001.11 in **a**, as well as Athena IFS simulations at T159 in **b**, T511 in **c**, and T1279 in **d**

Similar to the NP, the regression structures to NA PC1 of *vv*_250_ from both observations (Fig. 6a) and AGCM simulations (Fig. 6b–d) are characterized by a monopole center over the mean storm track background. The observed (Fig. 7a) and AGCM simulated regression patterns (Fig. 7b–d) related to NA PC2 consists of a latitudinal dipole structure, indicating a meridional shifting over the climatological-mean storm tracks. The regression of *vv*_250_ to NA PC3 (Supplementary Fig. 4) shows a zonal dipole structure. The structures of large magnitudes in observed and all simulated panels are restricted within the regions where the EOF analysis is calculated, suggesting that the shift of the storm track activities is largely a regional process. The simulated patterns have comparable percentages of the explained variance with those from observations, and there is no clear sensitivity of patterns to the model resolutions. The total variance explained by EOF1 varies around 17%, explained by EOF2 around 12%, and explained by EOF3 around 9% for both observations and simulations.

The insensitivity of the sectorial EOF patterns to model resolution can also be seen in the EOF correlation coefficients between simulations and observations in Table 1. All of the EOF correlation coefficients related to both NP and NA are very high and pass the significance test, but they do not have clear tendency with the resolution increases. On the other hand, the PC correlations between simulations and observations are all very small because of the high internal variability in both the models and in the observations.

### Connections of leading modes to atmospheric low-frequency variability

To examine the links between the storm track fluctuations and the atmospheric lower-frequency variability, we present the correlation patterns of the NH geopotential height with the corresponding PCs of *vv*_250_ in each sector. The monthly geopotential height fields adopted here are also on the 250 hPa level. Only areas with correlation passing the 95% statistical significance level are shaded. In practice, an examined time series may be time interdependent. Significant lag autocorrelations will reduce the degrees of freedom of the time series. In the correlation significance test of this study, the effective degrees of freedom are calculated using the Eq. (1) of Livezey and Chen (1983). The critical value for confidence level is determined by the Monte-Carlo experiments of 200 pairs of random series from standard normal distribution with the length equal to effective degrees of freedom of the examined pair of time series. The 95% confidence level of a two-tailed test corresponds to the fifth largest and the fifth smallest of the 200 correlation coefficients from the Monte-Carlo experiments. We also present the regression pattern of the geopotential height anomalies with the PCs in contours. The regression patterns are used to confirm the reliability of the corresponding correlation patterns.

**Fig. 8:**
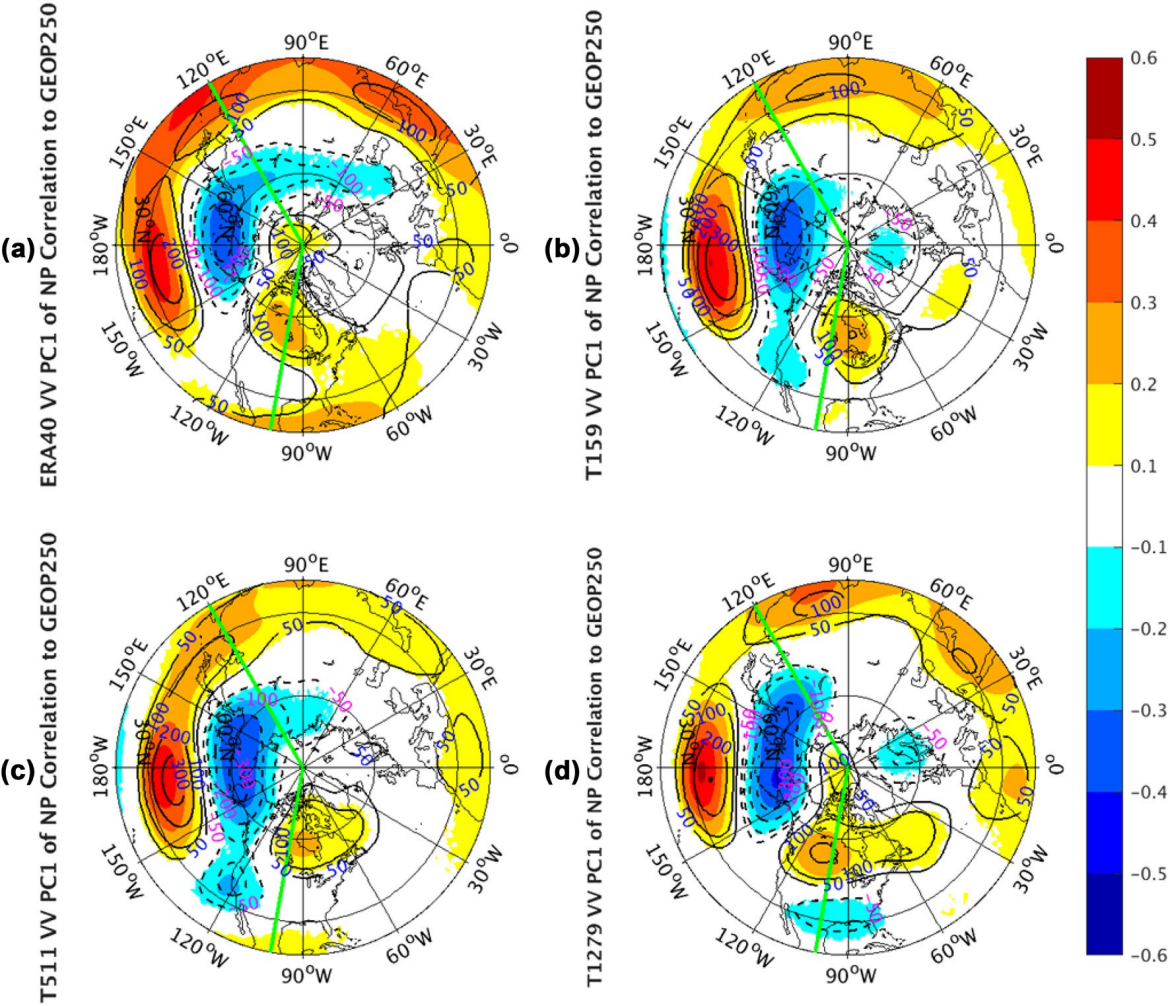
The correlation coefficients (shaded) and regression coefficients (contours) between PC1 of North Pacific sector, indicated by green lines, monthly averaged *vv*_250_ and the corresponding layer geopotential height fields from the ERA-40 reanalysis for 1960.12–2001.11 in **a**, as well as Athena IFS simulations at T159 in **b**, T511 in **c**, and T1279 in **d**. Only values of correlation coefficients that pass the significance test are displayed

Analyses about PC1 over the NP sector are shown in Fig. 8. The observed correlation panel (Fig. 8a) of geopotential height exhibits a spatial distribution similar to the Western Pacific (WP) pattern as described by Wallace and Gutzler (1981). With a north–south dipole of anomalies, its one center is located over the Kamchatka Peninsula and the other covers a portion of Southeast Asia and the western subtropical NP. The regression shape (contours) is able to well resemble this WP pattern, suggesting a reliable association between NP storm track activities and low frequency atmospheric variations. Note the regression patterns will not be repeatedly discussed in the rest of this paper if they can well match the corresponding correlation patterns. The connection of the NP EOF1 of storm track variability with West Pacific pattern is consistent with that derived by Wettstein and Wallace (2010) based on a regression of the WP index with the *vv*_300_ fields. The positive phase of the WP meridional dipole with positive anomalies in the southern lobe and negative anomalies in the northern lobe corresponds to anomalously strong westerly winds in the core of the mid-latitude jet stream. Physically, the strengthened westerly wind jet leads to increased storm-track activity (higher *vv*_250_) downstream. All AGCM simulations (Fig. 8b–d) are able to well reproduce this WP-storm track relationship, especially on the downstream effects of the strengthened or weakened westerly jet. Qualitatively, the model correlation patterns are not sensitive to the model resolutions.

**Fig. 9:**
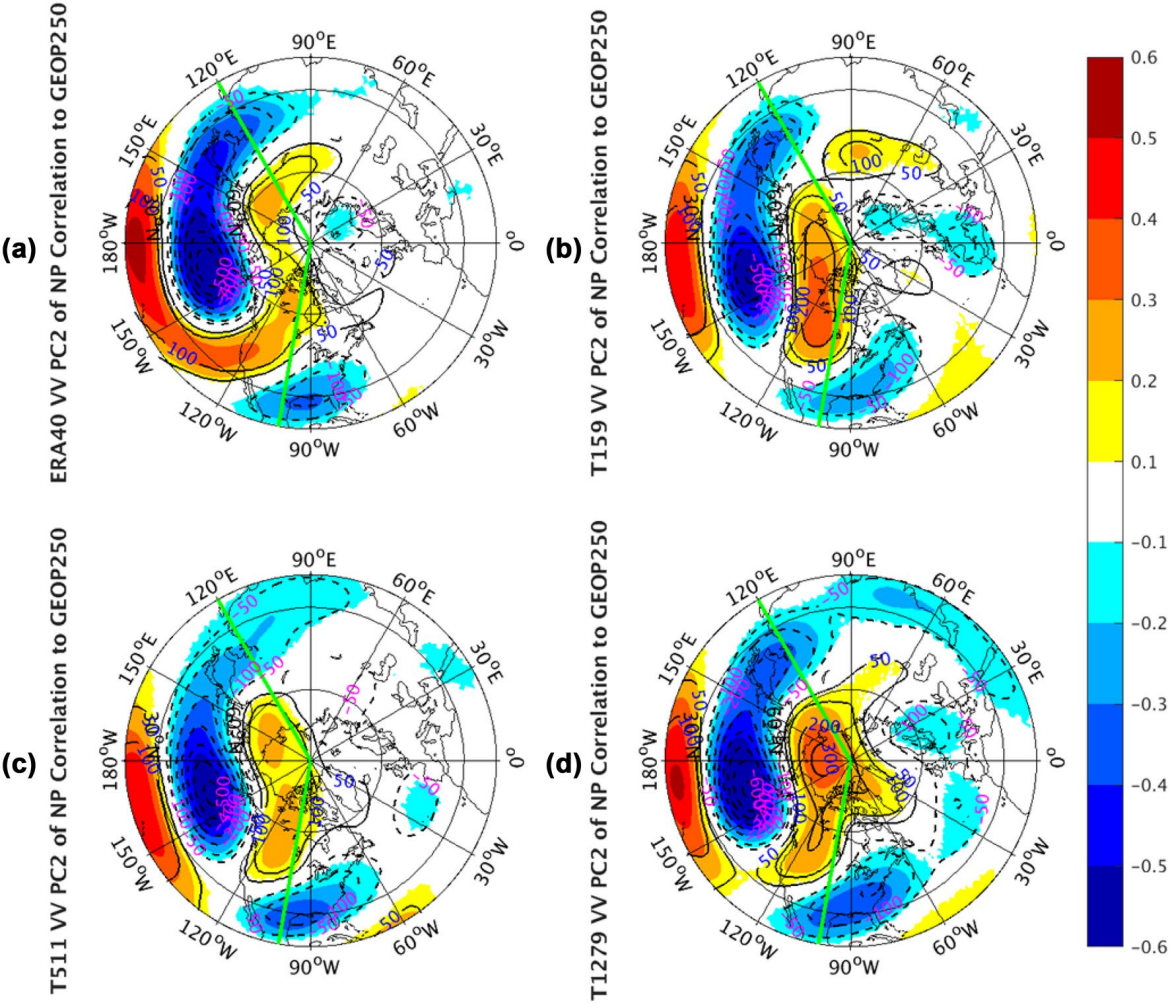
The correlation coefficients (shaded) and regression coefficients (contours) between PC2 of North Pacific sector, indicated by green lines, monthly averaged *vv*_250_ and the corresponding layer geopotential height fields from the ERA-40 reanalysis for 1960.12–2001.11 in **a**, as well as Athena IFS simulations at T159 in **b**, T511 in **c**, and T1279 in **d**. Only values of correlation coefficients that pass the significance test are displayed

The correlation structure of the observed 250 hPa geopotential height to PC2 of NP *vv*_250_ (Fig. 9a) in the NP sector seems to be localized, with negative correlations over the NP and southeastern United States, and with positive correlations in the vicinity of Hawaii. The AGCM simulated correlations at all three resolutions (Fig. 9b–d) can capture the major features of this observed pattern, but reproduce one more relatively weak positive center over the Intermountain Region of North America. This structure in simulations is similar to the Pacific/North American (PNA) pattern described by Wallace and Gutzler (1981). There are no substantial differences of this pattern in the simulations across all three resolutions. Actually in Cartesian coordinates (not shown), the geopotential height correlation pattern in ERA-40 exhibits a dipole oscillation structure over the NP and propagates toward two sides along the Northwest–Southeast direction. But the simulated 250 hPa geopotential height patterns (Fig. 9b–d), including the weaker positive center in Intermountain Region, display a well-known wave train which is described in Horel and Wallace (1981). The wave train emanates from the tropical central Pacific region, first propagating poleward to the NP, and then curving eastward to North American Intermountain Regions, and finally equatorward to the southeastern US along a great circle route.

**Fig. 10:**
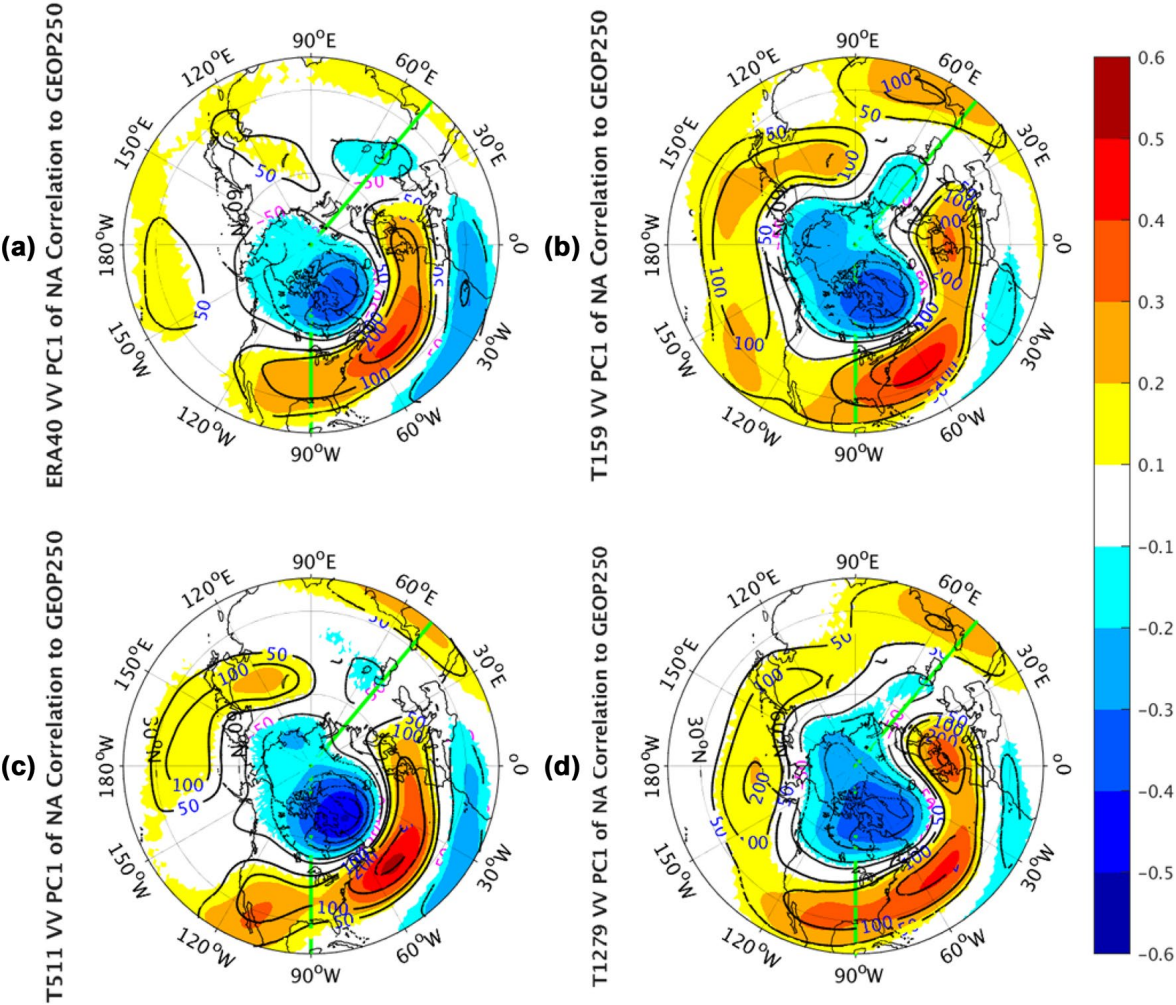
The correlation coefficients (shaded) and regression coefficients (contours) between PC1 of North Atlantic sector, indicated by green lines, monthly averaged *vv*_250_ and the corresponding layer geopotential height fields from the ERA-40 reanalysis for 1960.12–2001.11 in **a**, as well as Athena IFS simulations at T159 in **b**, T511 in **c**, and T1279 in **d**. Only values of correlation coefficients that pass the significance test are displayed

Similar analyses are also carried out in the NA sector. The correlation patterns of 250 hPa geopotential height to PC1 of NA *vv*_250_ are shown in Fig. 10. The observed correlation structure (Fig. 10a) shows a north–south dipole of anomalies over the extratropical NA, with one center located over Greenland and the other center of opposite sign spanning the central latitudes of the NA. This is a typical NA Oscillation (NAO) pattern, as defined in the original work by Walker and Bliss (1932), and consistent with the result in Ulbrich and Christoph (1999) that storm track activity is associated with a change of the NAO pattern. In fact, the observed geopotential height correlation structure indicates an association between the NA pulsing signal of the storm tracks and the NAO pattern, which can be seen as a response of the storm track activities to the lower-frequency jet stream variability in the jet exit regions (Wettstein and Wallace 2010). Curiously, the simulation at T511 (Fig. 10c) better reproduces these features than at the other two resolutions. The simulations at T159 (Fig. 10b) and T1279 (Fig. 10d) can generate the center over Greenland realistically, but produce the center with opposite sign over the central NA too far to the west. The differences in different resolution AGCM simulations are apparent, but there is no clear tendency to improve or downgrade the simulations with resolutions.

**Fig. 11:**
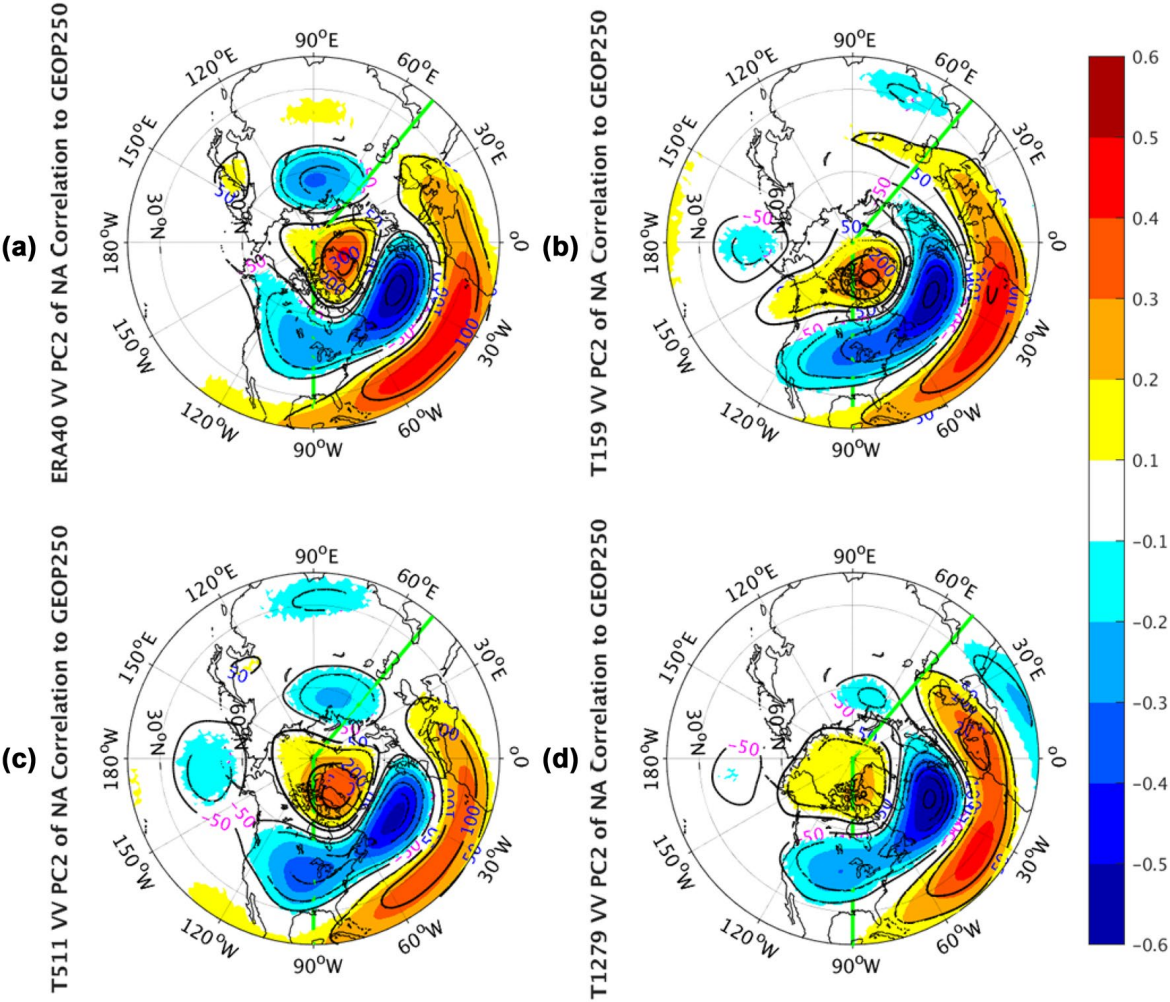
The correlation coefficients (shaded) and regression coefficients (contours) between PC2 of North Atlantic sector, indicated by green lines, monthly averaged *vv*_250_ and the corresponding layer geopotential height fields from the ERA-40 reanalysis for 1960.12–2001.11 in **a**, as well as Athena IFS simulations at T159 in **b**, T511 in **c**, and T1279 in **d**. Only values of correlation coefficients that pass the significance test are displayed

The geopotential height correlation shape (Fig. 11) to PC2 of NA *vv*_250_ in all panels demonstrates another north–south dipole of anomalies over the extratropical NA basin. This dipole pattern is structurally similar to NAO. Compared with NAO, it is located southeastward and in the nodal lines of the NAO pattern. Barnston and Livezey (1987) named it as East Atlantic (EA) Pattern, which is the second prominent mode of low-frequency variability over the NA. In fact, the correlation pattern regarding to the geopotential height field implies an association of the meridional displacement of the Atlantic storm track with the lower-frequency variability of the EA pattern. All AGCM simulations (Fig. 11b–d) can simulate the gross features of the EA pattern qualitatively, but there is somewhat mismatch of the subtropical center in all of these three simulated panels.

### Connections of leading modes to oceanic low-frequency variability

The oceanic fluctuations may also play an important role in the mid-latitude storm track activities. Correlation and regression analyses we discussed in the Sect. 4.2 are then applied between leading PCs of sectorial *vv*_250_ and the underlying SST. The calculation of effective degrees of freedom are also involved in the correlation significance test, and the Monte Carlo experiments are adopted to determine the critical values for the 95% confidence level.

**Fig. 12:**
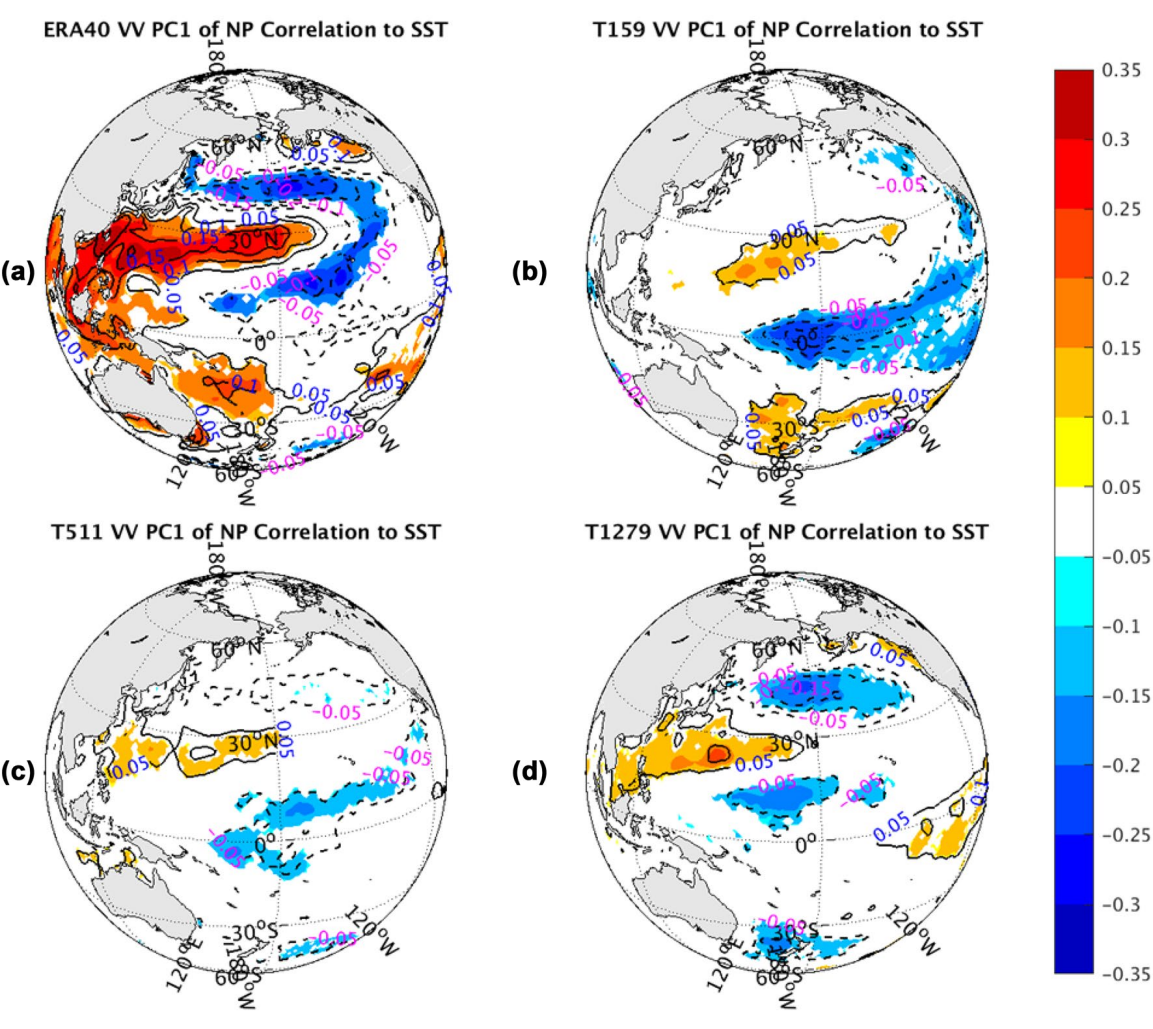
The correlation coefficients (shaded) and regression coefficients (contours) between PC1 of North Pacific sector monthly averaged *vv*_250_ and SST fields from the ERA-40 reanalysis for 1960.12–2001.11 in **a**, as well as Athena IFS simulations at T159 in **b**, T511 in **c**, and T1279 in **d**. Only values of correlation coefficients that pass the significance test are displayed

Figure 12 shows the correlation between NP PC1 of *vv*_250_ and NH SST, superimposed with the contours of the regression between them. The collocation pattern from observations (Fig. 12a) displays a positive center from East China Sea extension to central NP, surrounded by colder waters with a zonal U-shape in the NP. The positive anomalies also extend to the northern Indian Ocean and to the vicinity of the South Pacific Convergence Zone (SPCZ). The SST distribution in NP is similar to the negative phase of the NP Mode (NPM) as identified by Hartmann (2015, his Fig. 1c). This connection between storm track variations and the underlying SST oscillation can hardly be detected in the lower resolution (T159) AGCM simulation (Fig. 12b). With resolution getting finer, it is slightly better reproduced. The highest resolution run (T1279) (Fig. 12d) yields the best agreement with the observed one, but it is still far weaker than the reality.

**Fig. 13:**
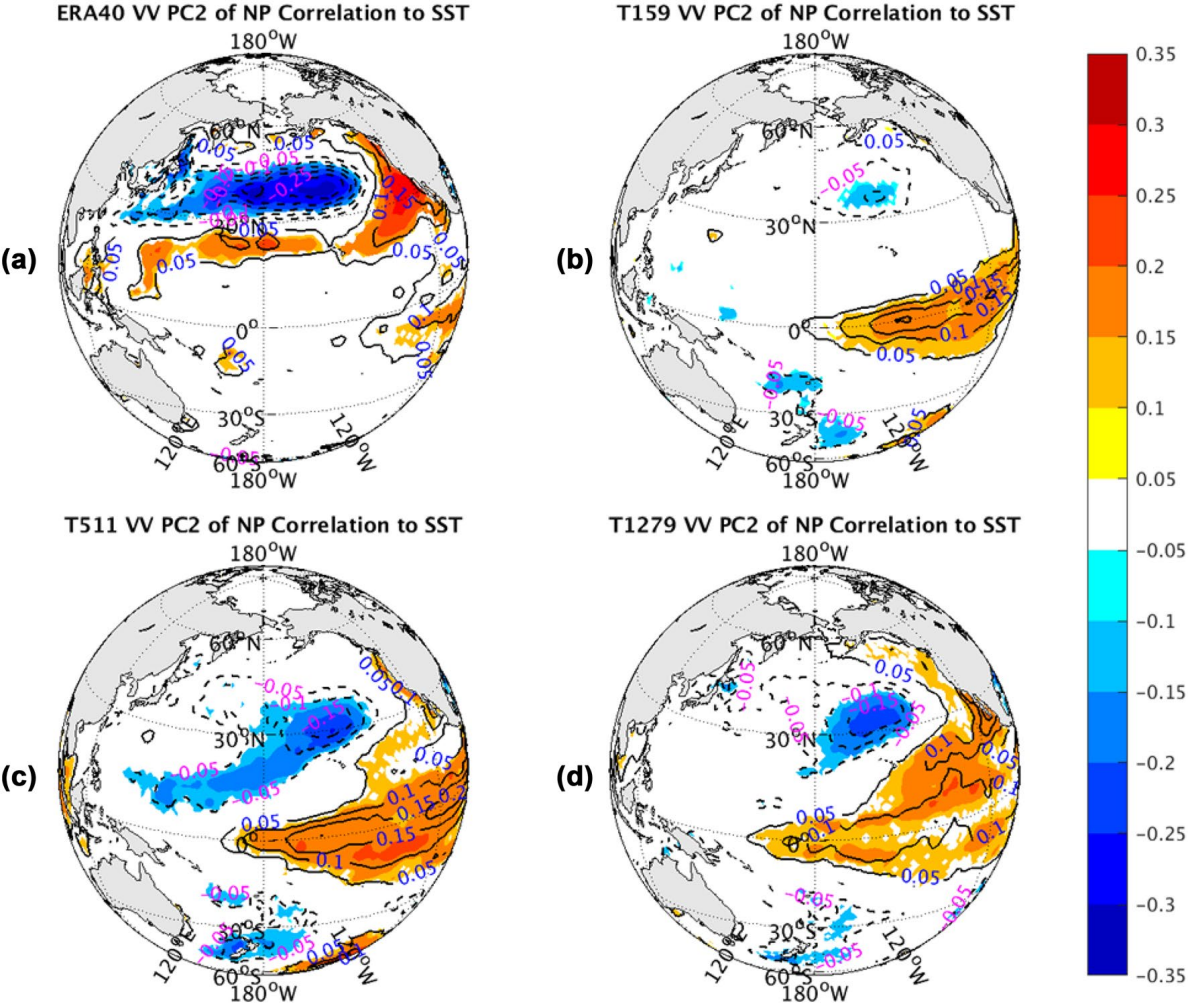
The correlation coefficients (shaded) and regression coefficients (contours) between PC2 of North Pacific sector monthly averaged *vv*_250_ and SST fields from the ERA-40 reanalysis for 1960.12–2001.11 in **a**, as well as Athena IFS simulations at T159 in **b**, T511 in **c**, and T1279 in **d**. Only values of correlation coefficients that pass the significance test are displayed

The structure associated with the correlations between SST and PC2 of NP *vv*_250_ are shown in Fig. 13. The observed correlation shape (Fig. 13a) exhibits a localized dipole structure in the NP along with some weak signals in the tropical Eastern Pacific. But the AGCM runs have distinct patterns. AGCM simulations (Fig. 13b–d) show warm waters with a horseshoe shape surrounding a core of cooler water, and positive anomalies over central and eastern equatorial Pacific, which describes the warm phase of El Niño-Southern Oscillation (ENSO) cycle. In fact, the tropical positive anomalies in the observations (Fig. 13a) also represent a part of the ENSO cycle. All resolution AGCM runs (Fig. 13b–d) can simulate this ENSO structure more or less, but cannot reproduce the observed NP dipole shape. Moreover, the simulated ENSO cycle is more apparent in high resolution runs. The features in the NP are hardly detected in the T159 simulation (Fig. 13b). As the resolution increases, the simulated tropical East Pacific SST anomalies tend to increase and the NP part of the ENSO pattern emerges.

The observed correlation pattern of SST anomalies to PC1 of NA *vv*_250_ forms a tripolar structure with a cold anomaly in the sub-polar region, a warm anomaly in the middle latitudes, and a cold sub-tropical anomaly (not shown). That suggests there are not only the essential relationships between EOF1 of NA *vv*_250_ and the atmospheric NAO pattern (Fig. 10), but also connections with the tripolar feature of underlying SST. The NAO links with the tripolar pattern of SST anomalies through regional changes has been revealed in Visbeck et al. (2003). However, none of the model simulations seem to reproduce this SST pattern (not shown).

The observed SST correlation pattern to PC2 of NA *vv*_250_ is also characterized by a tripolar structure of the SST anomalies. This tripolar pattern has a location displacement compared with the one related to PC1, and is located in the nodal lines of the other. The interactions between the underlying tripolar structures of NA SST and lower-frequency atmospheric variability aloft are revealed by Álvarez-García et al. (2011) that the NA tripolar of SST anomalies with 9-year period are associated with an atmospheric configuration resembling the EA pattern, whereas the 14-year period SST variations are likely related to the NAO pattern. Similar to correlation related to PC1 of NA *vv*_250_, the model simulated SST correlation patterns to PC2 of NA *vv*_250_ (not shown) either cannot capture the natural links between the SST tripolar anomalies and the NA latitudinal shift of *vv*_250_ (Fig. 7). As we will see in the next section, the coupled system can do better in this aspect.

## The effects of air–sea coupling and the potentially predictable fraction of *vv*_250_

In addition to the atmospheric internal interactions, the oceanic settings may also play an important role in the mid-latitude storm track activities. Two coupled CCSM runs (LRC01 and HRC06) are utilized to investigate the atmosphere–ocean coupling effects on the NH storm track interannual variability. Similar to the AGCM simulations in Figs. 2 and 3, the EOF analyses in the whole North Hemisphere domain demonstrate unsteady connections of storm track variations between NP and NA sectors with oceanic resolution changes in the CCSM simulations, and the magnitude of leading EOFs is significantly underestimated (not shown). The general features of the hemispheric EOF leading modes of NH *vv*_250_ from CCSM can be roughly represented in two individual sectors. For the sake of brevity, we will skip the analysis of the NH simulations as a whole, but conduct the diagnosis based on the individual sectors separately. Moreover, only the results from HRC06 run are displayed due to the unavailability of SST data from LRC01.

### The North Pacific sector

**Fig. 14:**
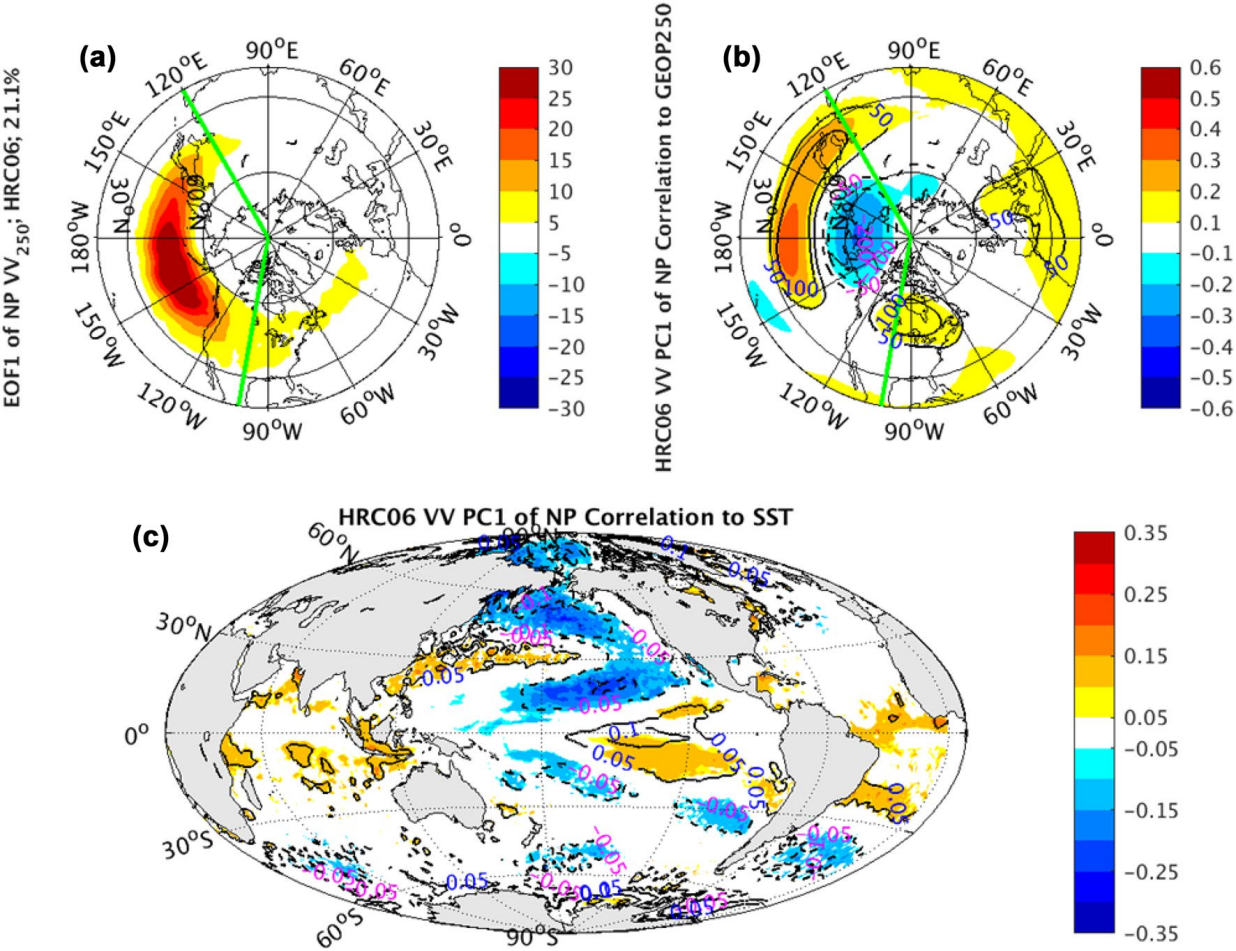
EOF1 (shaded) of North Pacific sector, indicated by green lines, monthly averaged *vv*_250_ superimposed by NH climatological mean *vv*_250_ (contours) in **a**, correlation coefficients (shaded) and regression coefficients (contours) between PC1 of North Pacific sector monthly averaged *vv*_250_ and the corresponding layer NH geopotential height fields in **b**, correlation coefficients (shaded) and regression coefficients (contours) between PC1 of North Pacific sector monthly averaged *vv*_250_ and global SST fields in **c**, from coupled CCSM simulations

The EOF1 of CCSM simulated HRC06 *vv*_250_ in the NP (Fig. 14a) sector is characterized by a strengthening or weakening of the climatological-mean storm tracks. This is consistent with the observations and simulations from ECMWF IFS atmospheric model in Fig. 4. However, comparison of the patterns from CCSM simulations (Fig. 14a) with observations and AGCM simulations (Fig. 4) illustrates a substantial underestimate in the magnitude of storm track variability in CCSM runs. The CCSM simulated mode looks like only half strength of observations and AGCM runs. This underestimate is not sensitive to the model oceanic resolutions. Since the leading EOFs of *vv*_250_ from LRC01 are very similar to the corresponding ones from HRC06 in both structure and magnitude, the LRC01 EOFs will not be shown in this study.

Consistent with observations (Fig. 8a), the geopotential height signatures of WP pattern (Fig. 14b), corresponding to PC1 of *vv*_250_ in the NP sector, is dominated by meridional dipoles with positive anomalies in the southern lobes and negative anomalies in the northern lobes. The strength of correlation coefficients (shaded) and regression coefficients (contours) in the CCSM HRC06 run (Fig. 14b) is much weaker than the observed WP pattern (Fig. 8a), even though the position of WP pattern is simulated successfully. The comparison of geopotential height and SST anomalous signatures between CCSM higher oceanic resolution and lower oceanic resolution runs will also not be illustrated in this study owning to the limitations of SST datasets.

The HRC06 SST anomalies corresponding to PC1 of NP *vv*_250_ (Fig. 14c) feature a strong NPM pattern in the NP along with a weak ENSO structure in the tropics. The weak ENSO shape, however, is not detected in the observations (Fig. 12a). We guess it is because the CCSM simulated pulsing (Fig. 14a) is slightly mixed by the ENSO forced storm track fluctuations. In AGCM runs, as discussed above, the NPM pattern is more or less reproduced with a substantial weaker magnitude than reality (Fig. 12). However, this underestimate is recovered in the coupled CCSM runs (Fig. 14c). A possible explanation to this substantial weakening of the SST variability in AGCM runs is that this EOF mode of *vv*_250_ in nature is a combination of strong atmospheric internal variations and weak external forcing from the ocean. The anomalous SST pattern in the observations (Fig. 12a) is mainly a response to the internal atmospheric variations plus the weak external SST forcing. Since the SST is prescribed in these model simulations, the SST response to the atmospheric internal variations is not produced, leading to the much weaker external forcing SST anomalies in the simulated panels (Fig. 12b–d). The comparison between atmospheric and coupled model simulations suggests that the SST NPM structure coupled with WP pattern, at least partly, work as forcing signals.

**Fig. 15:**
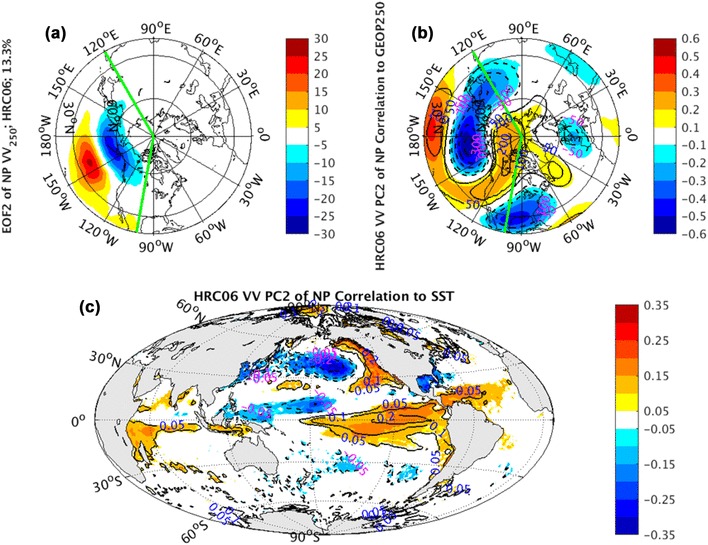
EOF2 (shaded) of North Pacific sector, indicated by green lines, monthly averaged *vv*_250_ superimposed by NH climatological mean *vv*_250_ (contours) in **a**, correlation coefficients (shaded) and regression coefficients (contours) between PC2 of North Pacific sector monthly averaged *vv*_250_ and the corresponding layer NH geopotential height fields in **b**, correlation coefficients (shaded) and regression coefficients (contours) between PC2 of North Pacific sector monthly averaged *vv*_250_ and global SST fields in **c**, from coupled CCSM simulations

The atmospheric features associated with PC2 of NP *vv*_250_, including the signatures of NP meridional dipole (e.g., EOF2) in Fig. 5 and the low-frequency atmospheric teleconnection of PNA-like pattern in Fig. 9 is structurally imitated by the coupled CCSM simulations (Fig. 15a, b). But their magnitudes are again underestimated significantly.

The observed NP dipole oscillation accompanied by weak ENSO structure in the tropics (Fig. 13a), coupling with the PNA pattern and linked with EOF2 of *vv*_250_ over NP sector, is also reproduced qualitatively in the coupled model simulations (Fig. 15c). Comparison of the SST patterns from observations (Fig. 13a) related to PC2, AGCM simulations (Fig. 13b–d), and coupled CCSM HRC06 simulation (Fig. 15c) associated with PC2 of *vv*_250_ shows a lack of outstanding localized dipole pattern in the AGCM runs. The absence of the dipole pattern in the AGCM runs also provides the evidence of atmospheric internal variability component for the NP meridional shift pattern (Fig. 5). On the other hand, since the SST response to atmospheric internal variations cannot be produced in the AGCM runs, the presence of ENSO pattern in both observation and AGCM simulations indicates an external forcing for this latitudinal shift pattern. In the observed correlation pattern of SST anomalies to PC2 of NP *vv*_250_, the external forcing pattern (ENSO) is intensified by the SST response to the internal atmospheric fluctuations, generating a prominent dipole structure in the North Central Pacific superimposing on the ENSO pattern. The ENSO forcing to storm track variations has been studied by Straus and Shukla (1997), Zhang and Held (1999), and Eichler and Higgins (2006). Our result is reminiscent of this connection that El Niño events drive the Pacific storm track shifts equatorward and downstream while La Nina drives in the opposite direction.

### The North Atlantic sector

**Fig. 16:**
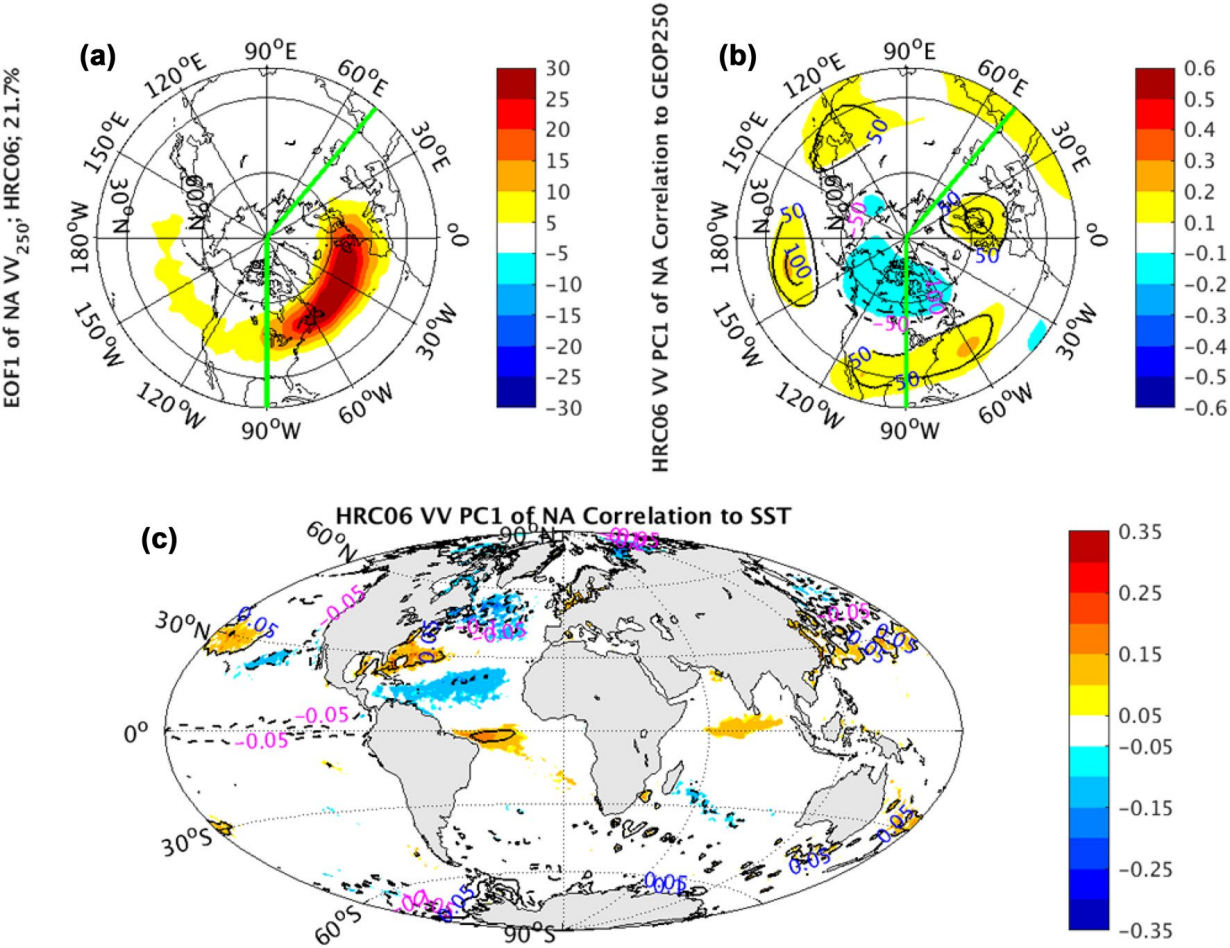
EOF1 (shaded) of North Atlantic sector, indicated by green lines, monthly averaged *vv*_250_ superimposed by NH climatological mean *vv*_250_ (contours) in **a**, correlation coefficients (shaded) and regression coefficients (contours) between PC1 of North Atlantic sector monthly averaged *vv*_250_ and the corresponding layer NH geopotential height fields in **b**, correlation coefficients (shaded) and regression coefficients (contours) between PC1 of North Atlantic sector monthly averaged *vv*_250_ and global SST fields in **c**, from coupled CCSM simulations

**Fig. 17:**
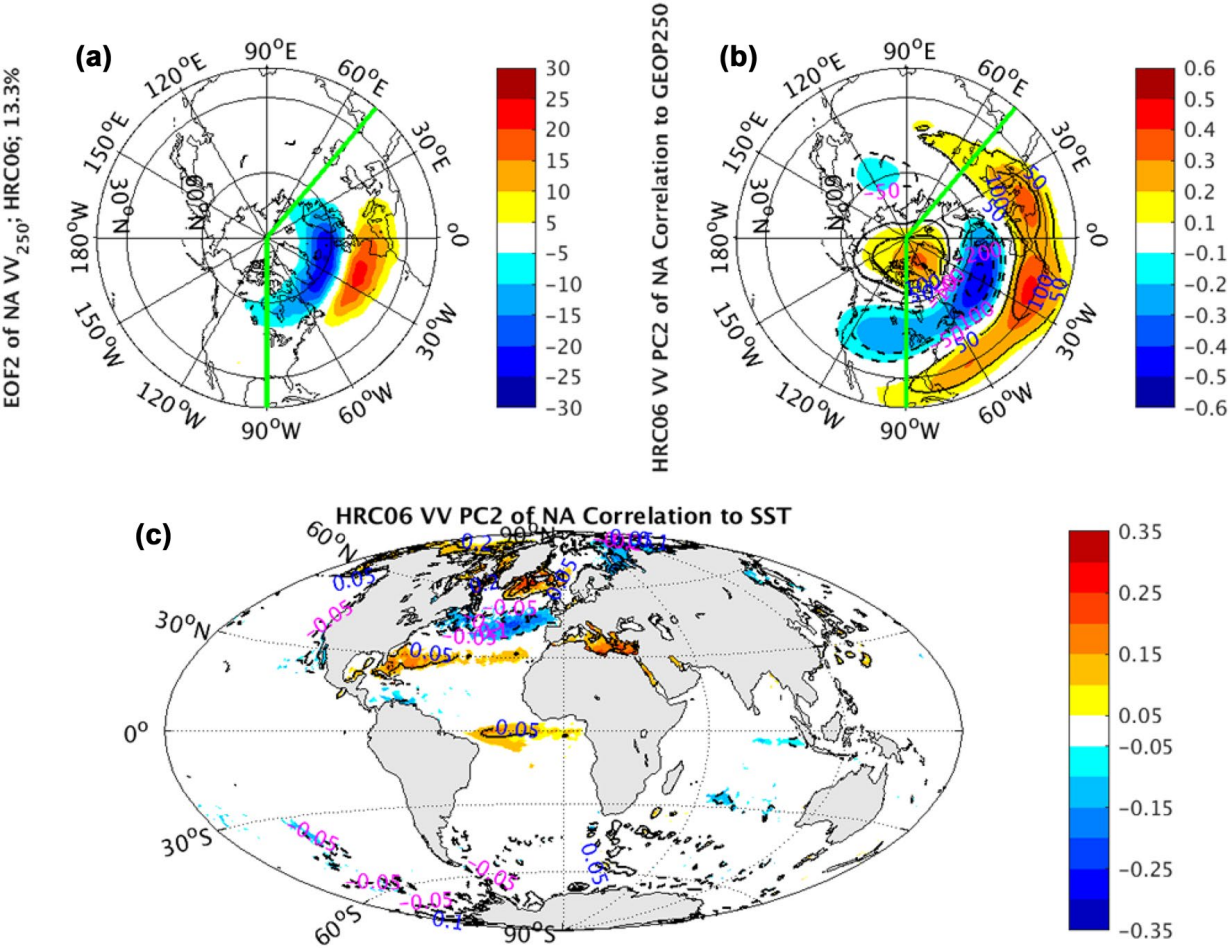
EOF2 (shaded) of North Atlantic sector, indicated by green lines, monthly averaged *vv*_250_ superimposed by NH climatological mean *vv*_250_ (contours) in **a**, correlation coefficients (shaded) and regression coefficients (contours) between PC2 of North Atlantic sector monthly averaged *vv*_250_ and the corresponding layer NH geopotential height fields in **b**, correlation coefficients (shaded) and regression coefficients (contours) between PC2 of North Atlantic sector monthly averaged *vv*_250_ and global SST fields in **c** from coupled CCSM simulations

Similar to NP sector, the CCSM simulated EOF1 of NA HRC06 *vv*_250_ (Fig. 16a) features pulsing, while EOF2 (Fig. 17a) is characterized as meridional shifting of the climatological mean storm tracks. Both leading EOFs in NA sector from CCSM runs are also dramatically underestimated.

In the geopotential height field, the NAO pattern from the observations (Fig. 10a) and AGCM simulations (Fig. 10b–d), corresponding to the PC1 of NA *vv*_250_, cannot be reproduced in the coupled CCSM simulations (Fig. 16b) realistically, where a different dipole structure over the North America continent shows up. Compared with the observed NAO pattern, it has a westward displacement and weaker correlation coefficients and regression coefficients. On the other hand, the EA pattern in the observations and AGCM simulations (Fig. 11), corresponding to the PC2 of NA *vv*_250_ and connected with the storm track meridional shifting, is structurally reproduced in this CCSM coupled system (Fig. 17b) qualitatively, but with considerably weakened regression coefficients.

The SST anomalies, correlated to the PC1 and PC2 of NA *vv*_250_ and linked to NAO and EA patterns respectively, show two distinct tripolars in the observations (not shown). They are all absent in the AGCM runs, but reproduced in the coupled CCSM simulations (Figs. 16c, 17c). The failure of AGCM reproducing the two NA tripolar patterns again can be explained as a response of the anomalous SST to the internal atmospheric variations. Compared the SST anomalies in AGCM runs with the SST anomalies in coupled CCSM runs, we can infer that the first two leading modes of NA *vv*_250_ are pure internal atmospheric variability.

### The predictable fraction of vv_250_

The storm track variability in the NP sector is partly forced by the underlying SST. The atmospheric fluctuations induced by the sustained SST forcing can persist on seasonal time scale and are thus more predictable (Shukla 1981). Using simple linear regression, the fraction of the potentially predictable components of the storm track interannual variability is estimated. The NP *vv*_250_ field and the leading PCs of the Pacific (120°E–270°E; 60°S–60°N) SST are used as the predictand and predictor, respectively. In this section, the first four leading SST modes (Supplementary Fig. 5) are investigated. Pacific SST EOF1 indicates the ENSO cycle; EOF2 represents the NPM, similar to the opposite phase of Fig. 1c in Hartman (2015); EOFs exhibits another NPM, as identified by Hartman (2015, his Fig. 1b); and EOF4, similar to Fig. 13a, is a localized NP dipole structure. Since the mean of *vv*_250_ has been subtracted, the interceptions for all grids in linear regression are zero. The predictable portion of *vv*_250_ is the ratio of the variance from the linear regression to the total variance of NP *vv*_250_. The predictable fraction forced by the first four SST modes in observations is 0.8%, 2.4%, 0.4%, and 0.7% in order. The predictable fraction for the four leading modes in Athena T159 is 1.1, 0.1, 0.3%, and 0.3; 1.1, 0.2, 0.2, and 0% in Athena T511; and 0.8, 0.2, 0.3, and 0.3% in Athena T1279, respectively. The total predictable fraction of the NP storm track variability in observations accounts for 4.4%, while it only has less than 2% of the total variance in all three AGCM resolution simulations. The fraction estimates of the NA SST forced storm track fluctuations will be skipped because they are merely from the atmospheric internal variability. We will neither discuss the predictable fraction of the storm track variability from CCSM coupled model simulations due to the substantial underestimates of its magnitude.

## Summary

Using outputs from atmospheric and coupled climate models, we have investigated the sensitivity of the simulated extratropical NH storm track interannual variability to the model horizontal resolution and air–sea coupling by performing EOF analysis on the monthly variance of high-pass filtered meridional winds, *vv*_250_, an indicator of storm track low-frequency fluctuations. The analyses based on the observations in the whole NH domain show an in-phase relationship of EOF1 and a seesaw relationship of EOF2 of *vv*_250_ over the NP branch and NA branch. These relationships are qualitatively simulated in the low-resolution run (T159) of the ECMWF IFS atmospheric model. However, the phase associations between NP and NA, in the hemispheric leading EOFs, change with the atmospheric resolution increases. The in-phase relationship gradually disappears, leaving a single monopole structure over the NP center in the highest resolution run (T1279). The seesaw relation also vanishes gradually with the pulsing signal over the NP branch turning into a dipole in the highest resolution run (T1279). This suggests both the in-phase relationship and seesaw relationship of storm track variability between two NH sectors are, consistent with Wettstein and Wallace (2010), not steady and predominantly “sectorial” rather than “hemispheric” in nature. The not-steady relationships are also represented in the coupled CCSM simulations with the oceanic resolution changes.

The first two leading EOFs of the NP *vv*_250_ from observations have similar structures with the corresponding ones from the NA sector. EOF1s of *vv*_250_ from both NP and NA sectors show a monopole center over the mean state storm track, and the EOF2s show that a dipole straddles the climatological mean storm track. The monopole center in the NP sector is linked to the atmospheric WP pattern and the oceanic NPM, and the dipole pattern linked to the atmospheric PNA-like pattern and the tropical oceanic El Nino pattern intensified by prominent localized dipole pattern in the NP. In the NA sector, the mono-sigh center is connected to atmospheric NAO pattern and oceanic tripolar pattern, and the dipole structure connected with EA pattern and another different oceanic tripolar pattern.

These sectorial features of the storm track variations are qualitatively reproduced in both the Atmospheric ECMWF runs and the coupled CCSM runs. The ECMWF runs generate comparable magnitudes of the leading EOFs with the observations, while the coupled CCSM runs significantly underestimate their magnitudes. The model simulated sectorial EOFs are not sensitive to either the atmospheric or the oceanic resolutions. The associations between sectorial leading EOFs and atmospheric low frequency variations are well reproduced by the ECMWF hindcasts. Except the NAO connected to the NA EOF1, the coupled CCSM simulations can also imitate the connections of basin-scale storm track variations to low frequency atmospheric fluctuations qualitatively, but with much weaker magnitude. These simulated relationships are not sensitive to the atmospheric or oceanic resolutions. Moreover, the relationships between the mid-latitude NP storm track variations and the oceanic forcing can be reproduced more or less in the ECMWF atmospheric model, and are gradually improved with atmospheric horizontal resolution increases. But the relationships of the NA storm track variability and the oceanic forcing failed to be generated in atmospheric ECMWF runs. The associations between the storm track variability over two individual sectors and the SST forcing, however, can be simulated in the CCSM coupled model. The sensitivity of the associations between storm track activities and oceanic low frequency variations to the air–sea coupling is an indication that the interaction between the ocean and atmosphere over NA is merely a response of SST to internal atmospheric variations, while over NP sector it is partly due to the external surface forcing. Actually, the external forced storm track variability is very small. It accounts for 4.4% of the total variance in the observations, and it is only less than 2% of the total in all AGCM simulations.

## Electronic supplementary material

Below is the link to the electronic supplementary material.

Supplementary material 1 (DOCX 689 KB)

## References

[CR1] Álvarez-García FJ, OrtizBevia MJ, CabosNarvaez WD (2011). On the structure and teleconnections of North Atlantic decadal variability. J Clim.

[CR2] Barnston AG, Livezey RE (1987). Classification, seasonality and persistence of low-frequency atmospheric circulation patterns. Mon Weather Rev.

[CR3] Blackmon ML (1976). A climatological spectral study of the 500mb geopotential height of the Northern Hemisphere. J Atmos Sci.

[CR4] Chang EKM (2004). Are the Northern Hemisphere winter storm tracks significantly correlated?. J Clim.

[CR5] Chang EKM (2009). Are band-pass variance statistics useful measures of storm track activity? Re-examining storm track variability associated with the NAO using multiple storm track measures. Clim Dyn.

[CR6] Chang EKM, Fu Y (2002). Interdecadal variations in Northern Hemisphere winter storm track intensity. J Clim.

[CR7] Dirmeyer PA, Cash B, Kinter IIIA, Jung JL, Marx TL, Satoh M, Stan C, Tomita H, Towers P, Wedi N, Achuthavarier D, Adams JM, Altshule EL, Huang B, Jin EK, Manganello J (2012). Simulating the hydrologic diurnal cycle in global climate models: resolution versus parameterization. Clim Dyn.

[CR8] Eichler T, Higgins W (2006). Climatology and ENSO-related variability of North American extratropical cyclone activity. J Clim.

[CR9] Feng X, Huang B, Kirtman BP (2017). A multi-model analysis of the resolution influence on precipitation climatology in Gulf Stream region. Clim Dyn.

[CR10] Graff LS, LaCasce JH (2012). Changes in the extratropical storm tracks in response to changes in SST in an AGCM. J Clim.

[CR11] Grise KM, Son S-W, Gyakum JR (2013). Intraseasonal and interannual variability in North American storm tracks and its relationship to equatorial Pacific variability. Mon Weather Rev.

[CR12] Hartmann DL (2015). Pacific sea surface temperature and the winter of 2014. Geophys Res Lett.

[CR13] Hawcroft MK, Shaffrey LC, Dacre HF (2012). How much Northern Hemisphere precipitation is associated with extra-tropical cyclones?. Geophys Res Lett.

[CR14] Horel JD, Wallace JM (1981). Planetary-scale atmospheric phenomena associated with the Southern Oscillation. Mon Weather Rev.

[CR15] Kinter J, Cash B (2013). Revolutionizing climate modeling with Project Athena: a multi-institutional, international collaboration. Bull Am Meteorol Soc.

[CR16] Kirtman BP (2012). Impact of ocean model resolution on CCSM climate simulations. Clim Dyn.

[CR17] Lau N-C (1988). Variability of the observed midlatitude storm tracks in relation to low-frequency changes in the circulation pattern. J Atmos Sci.

[CR18] Leckebusch GC, Ulbrich U (2004). On the relationship between cyclones and extreme windstorm events over Europe under climate change. Glob Planet Change.

[CR19] Livezey RE, Chen WY (1983). Statistical field significance and its determination by Monte Carlo techniques. Mon Weather Rev.

[CR20] North GR, Bell TL, Cahalan RF, Moeng FJ (1982). Sampling errors in the estimation of empirical orthogonal functions. Mon Weather Rev.

[CR21] Pfahl S, Wernli H (2012). Quantifying the relevance of cyclones for precipitation extremes. J Clim.

[CR22] Pinto JG, Froehlich EL, Leckebusch GC, Ulbrich U (2007). Changing European storm loss potentials under modified climate conditions according to ensemble simulations of the ECHAM5/MPI-OM1 GCM. Nat Hazards Earth Syst Sci.

[CR23] Schwierz C, Kollner-Heck P, Mutter EZ, Bresch DN, Vidale P-L, Wild M, Schar C (2010). Modelling European winter wind storm losses in current and future climate. Clim Change.

[CR24] Shukla J (1981). Dynamical predictability of monthly means. J Atmos Sci.

[CR25] Shukla J, Hagedorn R (2009). Strategies: revolution in climate prediction is both necessary and possible: a declaration at the World Modeling Summit for Climate Prediction. Bull Am Meteorol Soc.

[CR26] Straus DM, Shukla J (1997). Variations of midlatitude transient dynamics associated with ENSO. J Atmos Sci.

[CR27] Ulbrich U, Christoph M (1999). A shift of the NAO and increasing storm track activity over Europe due to anthropogenic greenhouse gas forcing. Clim Dyn.

[CR28] Uppala SM (2005). The ERA-40 re-analysis. Q J R Meteorol Soc.

[CR29] Visbeck M, Chassignet E, Curry R, Delworth T, Dickson B, Krahmann G, Hurrell J, Kushnir Y, Ottersen G, Visbeck M (2003). The ocean’s response to North Atlantic Oscillation variability. The North Atlantic Oscillation.

[CR30] Walker GT, Bliss EW (1932). World weather V. Mem R Meteorol Soc.

[CR31] Wallace JM, Gutzler DS (1981). Teleconnections in the geopotential height field during the Northern Hemisphere winter. Mon Weather Rev.

[CR32] Wettstein JJ, Wallace JM (2010). Observed patterns of month-to-month storm track variability and their relationship to the background flow. J Atmos Sci.

[CR33] Yang X, Chang EKM (2006). Variability of the Southern Hemisphere winter split flow: a case of two-way reinforcement between mean flow and eddy anomalies. J Atmos Sci.

[CR34] Yang X, Chang EKM (2007). Eddy–zonal flow feedback in the Southern Hemisphere winter and summer. J Atmos Sci.

[CR35] Yang X (2015). Seasonal predictability of extratropical storm tracks in GFDL’s high-resolution climate prediction model. J Clim.

[CR36] Zhang Y, Held IM (1999). A linear stochastic model of a GCM’s midlatitude storm tracks. J Atmos Sci.

