# Supplementary material for: An examination of the Northern Hemisphere mid-latitude storm track interannual variability simulated by climate models—sensitivity to model resolution and coupling
Source: Clim Dyn. 2018 Aug 4;52(7):4247–68. doi: 10.1007/s00382-018-4378-x (PMC6445401; doi:10.1007/s00382-018-4378-x)
Supplement: Supplementary file 1 — Supplementary material 1 (DOCX 689 KB) [file 382_2018_4378_MOESM1_ESM.docx]

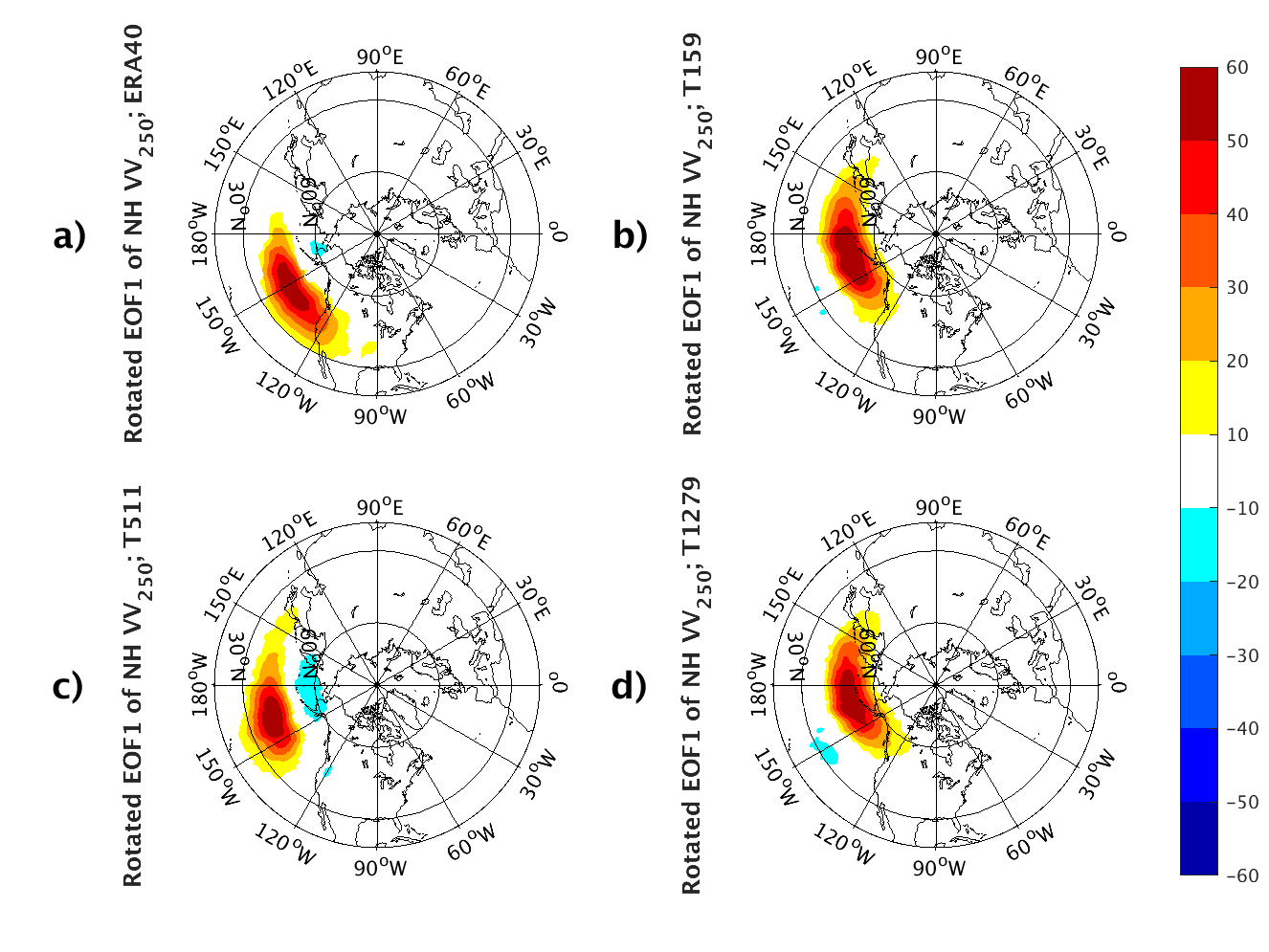


Supplementary Figure 1 Rotated EOF1 of NH monthly averaged *vv*_250_ for 1960.12–2001.11 from ERA40 in (a), T159 in b), T511 in c), and T1279 in d).


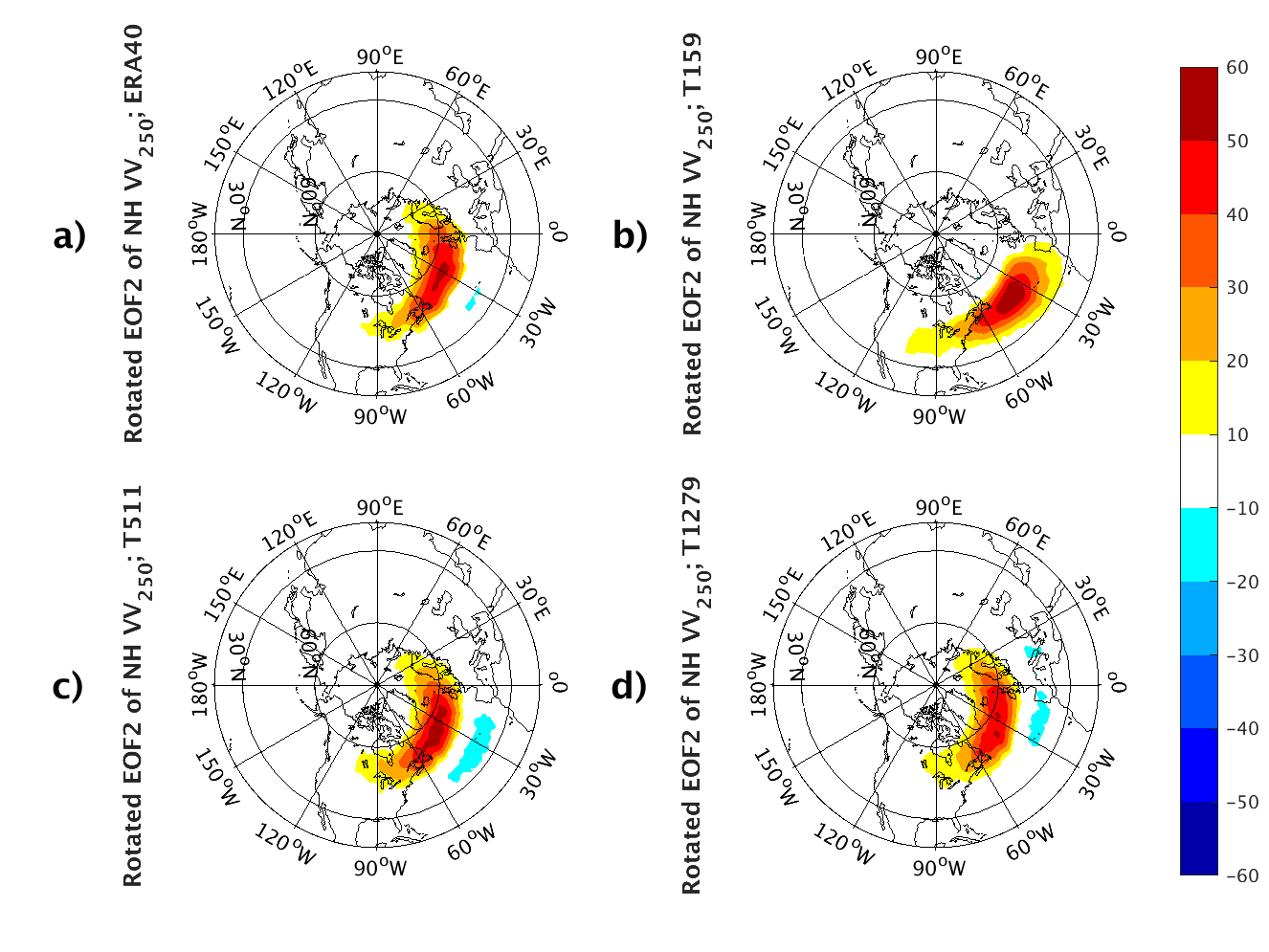


Supplementary Figure 2 Rotated EOF2 of NH monthly averaged *vv*_250_ for 1960.12–2001.11 from ERA40 in (a), T159 in b), T511 in c), and T1279 in d).


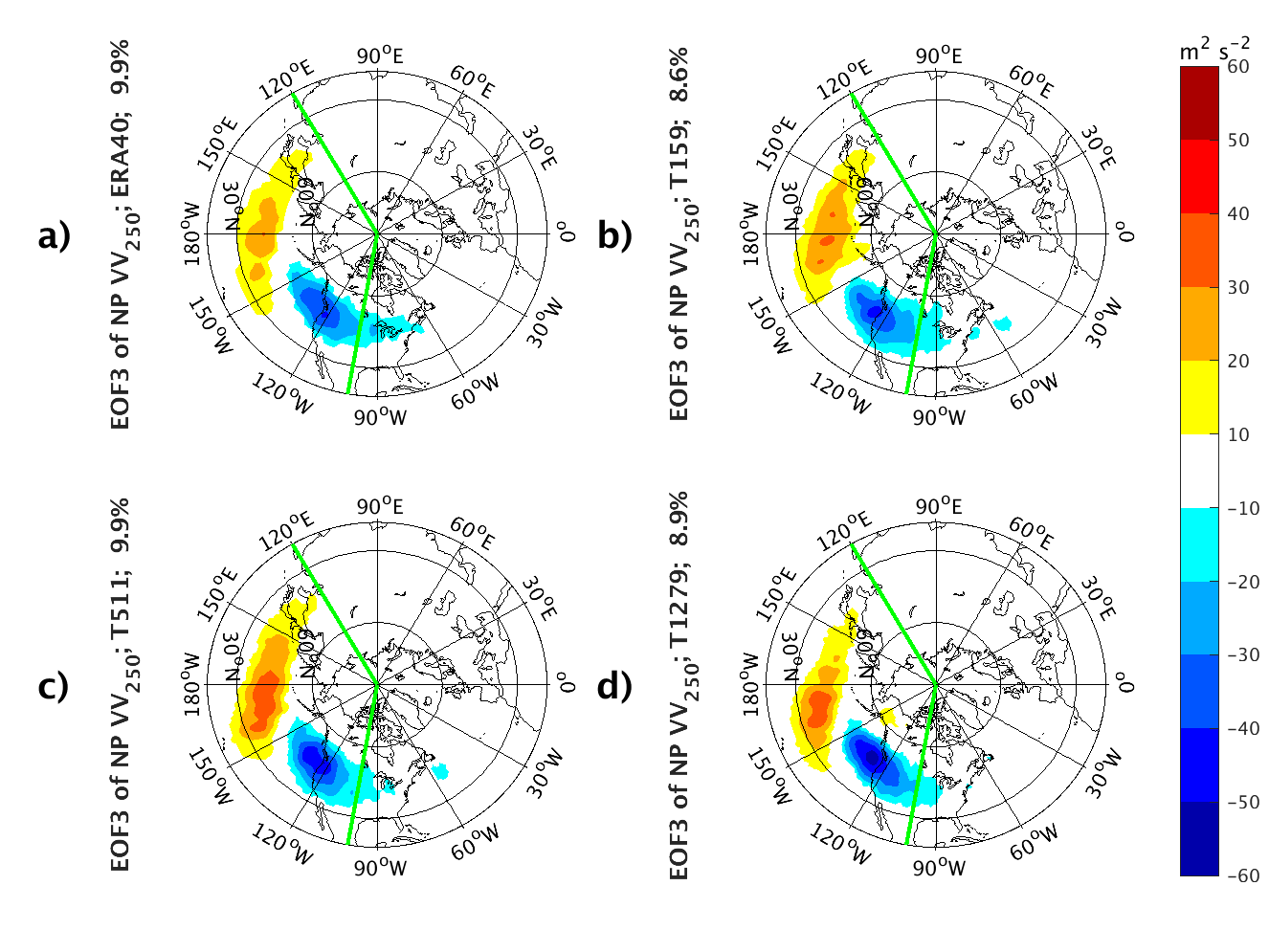


Supplementary Figure 3 EOF3 of North Pacific sector, indicated by green lines, monthly averaged *vv*_250_ from the ERA-40 reanalysis for 1960.12–2001.11 in panel (a), as well as Athena IFS simulations at T159 in panel (b), T511 in panel (c), and T1279 in panel (d).


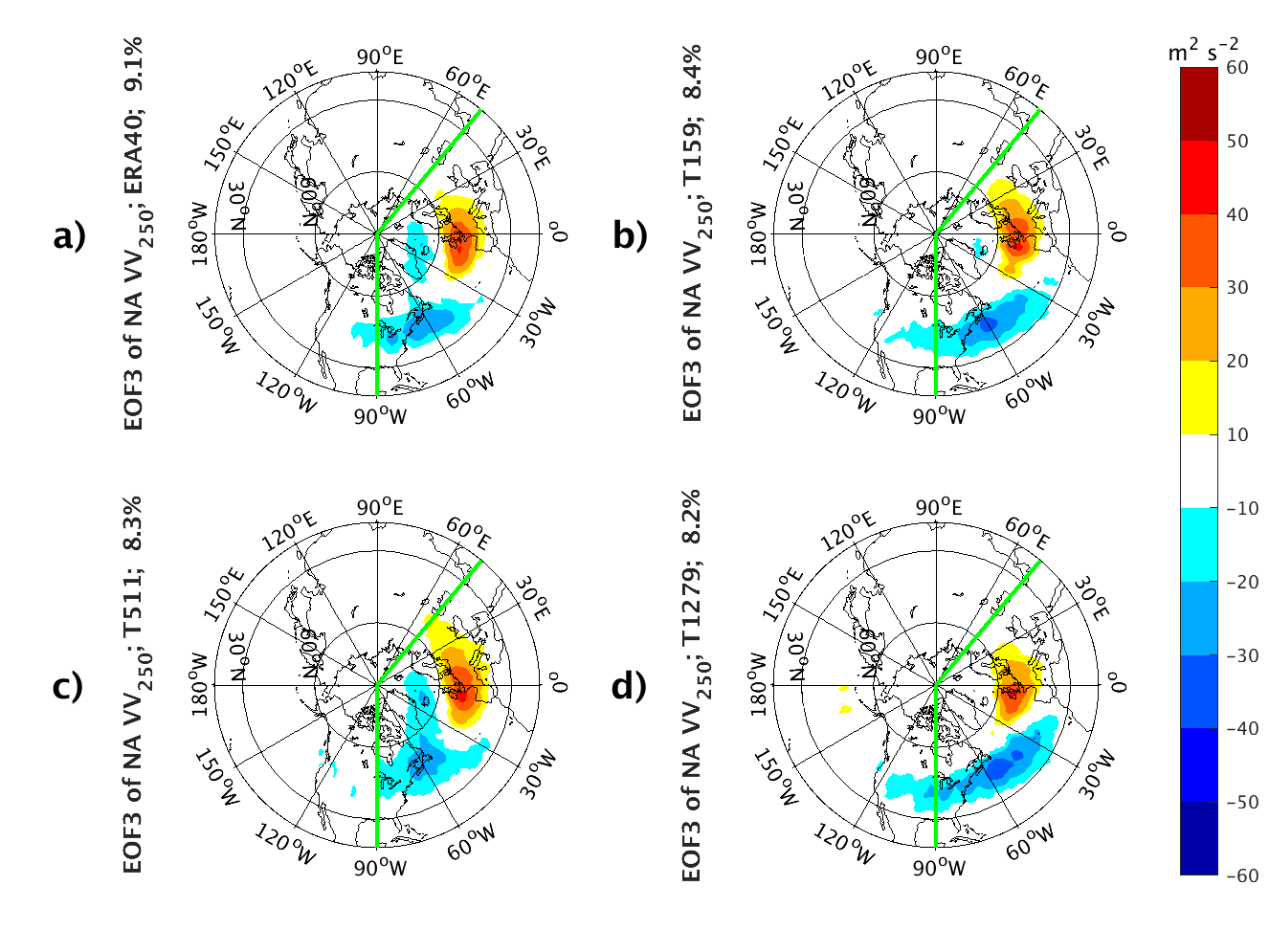


Supplementary Figure 4 EOF3 of North Atlantic sector, indicated by green lines, monthly averaged *vv*_250_ from the ERA-40 reanalysis for 1960.12–2001.11 in panel (a), as well as Athena IFS simulations at T159 in panel (b), T511 in panel (c), and T1279 in panel (d).


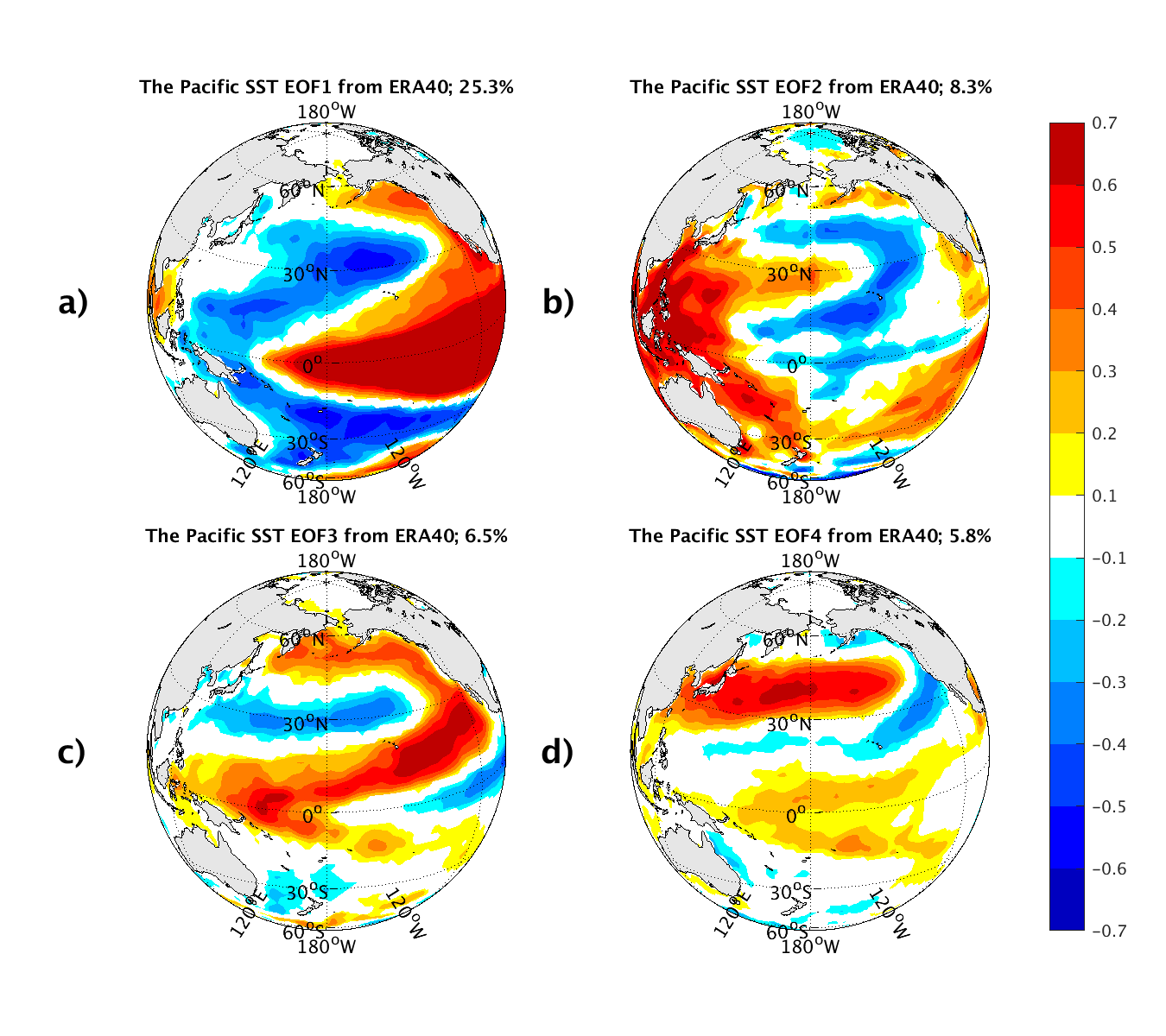


Supplementary Figure 5 First four leading EOFs of the Pacific monthly averaged SST from ERA40 for 1960.12–2001.11.
